# Functional characterization of D-type cyclins involved in cell division in rice

**DOI:** 10.1186/s12870-024-04828-9

**Published:** 2024-03-01

**Authors:** Zhishu Jiang, Xin Wang, Zhiwei Zhou, Limei Peng, Xiaoli Lin, Xiaowei Luo, Yongping Song, Huaying Ning, Cong Gan, Xiaopeng He, Changlan Zhu, Linjuan Ouyang, Dahu Zhou, Yicong Cai, Jie Xu, Haohua He, Yantong Liu

**Affiliations:** 1 Key Laboratory of Crop Physiology, Ecology, and Genetic Breeding of the Ministry of Education, Jiangxi Agricultural University, Nanchang, Jiangxi China; 2 Jiangxi Province Forest Resources Protection Center, Nanchang, 330008 Jiangxi China

**Keywords:** Rice, D-type cyclin, Cell cycle, Gene expression, Protein interaction

## Abstract

**Background:**

D-type cyclins (CYCD) regulate the cell cycle G_1_/S transition and are thus closely involved in cell cycle progression. However, little is known about their functions in rice.

**Results:**

We identified 14 *CYCD* genes in the rice genome and confirmed the presence of characteristic cyclin domains in each. The expression of the *OsCYCD* genes in different tissues was investigated. Most *OsCYCD* genes were expressed at least in one of the analyzed tissues, with varying degrees of expression. Ten OsCYCD proteins could interact with both retinoblastoma-related protein (RBR) and A-type cyclin-dependent kinases (CDKA) forming holistic complexes, while OsCYCD3;1, OsCYCD6;1, and OsCYCD7;1 bound only one component, and OsCYCD4;2 bound to neither protein. Interestingly, all OsCYCD genes except OsCYCD7;1, were able to induce tobacco pavement cells to re-enter mitosis with different efficiencies. Transgenic rice plants overexpressing *OsCYCD2;2*, *OsCYCD6;1*, and *OsCYCD7;1* (which induced cell division in tobacco with high-, low-, and zero-efficiency, respectively) were created. Higher levels of cell division were observed in both the stomatal lineage and epidermal cells of the *OsCYCD2;2-* and *OsCYCD6;1*-overexpressing plants, with lower levels seen in *OsCYCD7;1*-overexpressing plants.

**Conclusions:**

The distinct expression patterns and varying effects on the cell cycle suggest different functions for the various OsCYCD proteins. Our findings will enhance understanding of the CYCD family in rice and provide a preliminary foundation for the future functional verification of these genes.

**Supplementary Information:**

The online version contains supplementary material available at 10.1186/s12870-024-04828-9.

## Background

The control of morphogenesis during development requires coordination between cell proliferation, cell growth, and cell differentiation. As in any multicellular organism, cell proliferation is a key component of plant growth. Progression of the eukaryotic cell cycle is regulated by the Ser/Thr kinases, in particular, those of the cyclin-dependent kinase (CDK) family [1]. CDK complexes are formed by the association of catalytic kinase domains with regulatory cyclin subunits, with specific CDKs interacting with specific cyclin subsets to regulate particular phases of the cell cycle [2–4]. Cyclins thus control the activity of CDKs, as well as contribute to the subcellular locations, substrate specificities, and overall stability of CDK-cyclin complexes [5–7].

Cyclin genes have been identified in numerous organisms, with each organism containing a wide variety. Several cyclin subclasses are recognized, according to expression patterns, sequence similarities, and activities during the cell cycle [8]. At least 22 cyclins have been identified in humans; these are classified into 13 types, such as A- to I-type, K-, L-, and T-type, and UNG2 (Uracil DNA Glycosylase 2) proteins [9, 10]. Numerous cyclins are also found in plants, where they tend to be encoded by more genes than seen in animals, with even the small *Arabidopsis* genome containing at least 50 genes while rice has a minimum of 49 cyclins [8, 11]. As shown by phylogenetic analyses, these can all be classified as A- to D-type, H-, L-, T-, P-, J18-, and SOLO DANCER (SDS)-types. The A-, B-, and D-type cyclins (CYCD) appear to play major roles in cell cycle control [12], particularly at the main transition points of the G_1_/S and G_2_/M boundaries [9]. Both A- and B-type cyclins are expressed during the S and M phases, where they regulate DNA replication, the G_2_/M transition, and mitosis, whereas the CYCD proteins control the G_1_/S transition [5].

The CYCD-CDK/Rb/E2F/DP pathway at the entry to G_1_/S shows strong conservation in higher eukaryotes. CYCD proteins form active complexes with different CDKs (CDK4 and CDK6 in animals and CDKA in plants), leading to the phosphorylation of the retinoblastoma (Rb) protein and its subsequent binding to E2F/DP transcription factors, heterodimers that bind to E2F sites in the promoter regions of various genes, many of which are involved in both cell cycle progression and cell growth. The presence of Rb can recruit histone deacetylase activity to the bound E2F proteins, preventing transcription of the regulated genes. The association between E2Fs and Rb is blocked by Rb phosphorylation, allowing the transcription of the E2F-regulated genes and leading to S-phase entry [12–14]. Despite the conservation of CYCD functions, the sequence similarities between mammalian and plant CYCDs are only between 9 and 14% in the cyclin core region, although they all contain the Rb-binding motif LxCxE (where x represents any amino acid) [15–17]. The expression of CYCD proteins has been demonstrated to promote cell division in plants. For example, overexpression of *CYCD1;1* from *Antirrhinum majus* in tobacco BY-2 cells promoted entry into S phase and mitosis, and was linked with CDK activity associated with histone H1 and Rb proteins [18], while overexpression of *Arabidopsis CYCD2;1* promoted meristematic division in tobacco plants through decreasing the duration of the G_1_ phase [19]. Similarly, constitutive expression of *CYCD3;1* in *Arabidopsis* decreased the numbers of cells in G_1_ while extending the duration of G_2_, suggesting a dominant role for CYCD3;1 in the G_1_/S transition [20]. We previously observed that transient expression of *AtCYCD3;1* and *AtCYCD4;2* in tobacco leaf cells promoted the re-entry of pavement cells into cell division [21].

The CYCD members in plants are divided into seven subclasses (CYCD1–7). *Arabidopsis* contains 10 CYCD genes [22, 23]. Their activities in cell division have been well documented. Overexpression of both *AtCYCD2;1* and *AtCYCD1;1* led to rapid increases in the numbers of cycling cells, particularly in the central root region, accelerating germination and radicle emergence [24]. In contrast, *AtCYCD3;1* did not stimulate these processes and in fact, markedly delayed germination [24]. However, overexpression of *AtCYCD3;1* resulted in marked changes in the leaf architecture, including an absence of separate spongy and palisade mesophyll layers, with the peridermis consisting of numerous poorly differentiated polygonal cells [25]. Overexpression of *AtCYCD3;2* resulted in propeller-like rosettes with narrow dome-shaped leaves, plants overexpressing *AtCYCD3;3* showed reduced growth with no changes in the leaves [26]. Overexpression of *AtCYCD4* was found to enhance cell division in non-protruding cell files in the upper hypocotyl regions associated with stomata formation [27]. Interestingly, AtCYCD6;1 and AtCYCD7;1 seemed to limit the essential formative divisions during development [28, 29].

There are 14 *CYCD* genes in rice and, similar to *Arabidopsis*, they have been grouped into seven subfamilies [11]. However, despite the identification of these rice CYCDs, little is known of their functions in development and the cell cycle. The only report, to date, is that OsCYCD3;2 promotes branch formation through the regulation of cell division, thus maintaining the functions of the axillary meristem and SAM, with *oscycd3;2* plants showing significantly fewer branches than the wild-type plants [30]. Here, 14 *CYCD* genes were identified in rice and their gene structures, conserved motifs, and phylogenetic relationships were analyzed. Their interactions with OsCDKAs and OsRBRs were investigated using yeast two-hybrid and bimolecular fluorescence complementation assays. The role of these OsCYCDs in inducing cell division was evaluated using a mitotic model system in tobacco. Analysis of tissue expression and the effects of overexpression was performed to determine the roles of OsCYCDs in cell division in rice. The findings provide new insights and information on the functions of OsCYCDs in rice.

## Results

### **Expression profiles of*OsCYCDs*genes in various tissues**

**Fig. 1:**
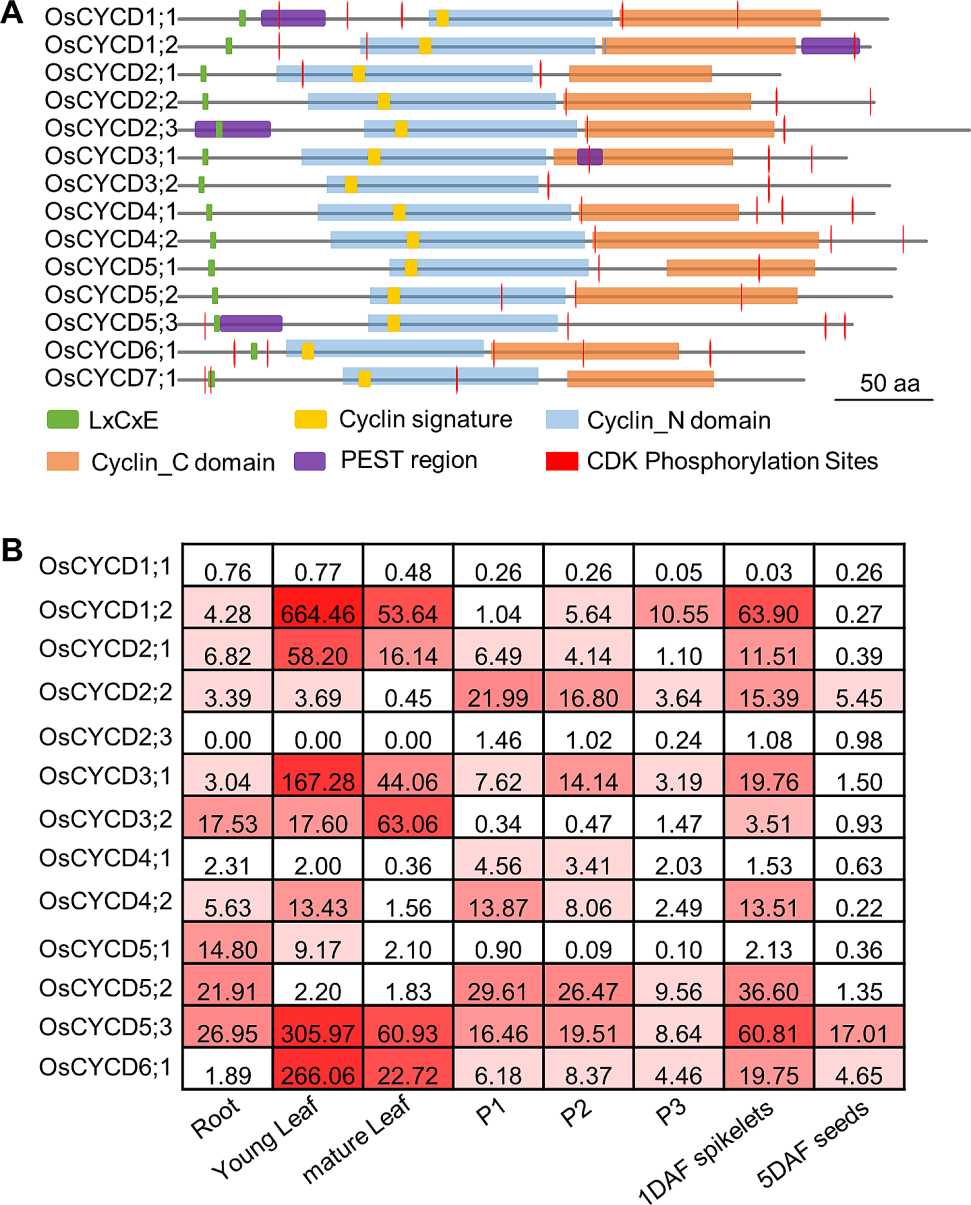
The domain structure and expression pattern of the 14 *OsCYCD* genes. **(A)** The domain structure of OsCYCD proteins. All OsCYCDs contain both cyclin_N and cyclin_C domains, cyclin signature, LxCxE, and CDK phosphorylation sites. OsCYCD1;1, OsCYCD1;2, OsCYCD2;3, OsCYCD3;1 and OsCYCD5;3 contain typical PEST region. **(B)** The expression pattern of *OsCYCD* genes in rice tissues, including 2-week-seedling roots and leaves (young leaf), heading-stage flag leaves (mature leaf), young inflorescences (< 2 cm, 2–5 cm, > 5 cm; P1, P2, and P3, respectively), spikelets (1 day after fertilization [DAF]), and 5 DAF seeds. The value represent 2^−ΔCT^×1000, with the UBQ gene as a control. The data presented are the means of three biological replicates

Fourteen *CYCD* genes were identified by keyword searches, BLAST searches, and sequence alignments in the rice genome. These genes were isolated from rice tissues using specific primers, confirming the presence of the predicted transcripts (data not shown). The locus IDs of the OsCYCDs are listed in Table. S5. All 14 rice CYCD proteins contained both cyclin N and cyclin C domains, the cyclin signature and the LxCxE motif, as well as CDK phosphorylation sites, which are important for CYCD function (Fig. 1A). Five proteins, OsCYCD1;1, OsCYCD1;2, OsCYCD2;3, OsCYCD3;1, and OsCYCD5;3, contained a typical PEST region which is responsible for the rapid degradation of CYCDs by ubiquitin-mediated proteolysis (Fig. 1A); although some atypical or poorly conserved PEST regions were also identified in other members [31], suggesting that different OsCYCDs might have different lifespans resulting in different effects on the cell cycle. Alignments of the predicted protein sequences and the phylogenetic trees constructed of both full sequences and the cyclin_N domains of CYCDs from rice, maize, and *Arabidopsis* are shown in Fig. S1. It was found that OsCYCDs shared greater sequence similarity with ZmCYCDs than with AtCYCDs, revealing their evolutionary relationships to a certain degree.

Using qRT-PCR, we analyzed the expression of *OsCYCD* genes in various tissues, including two-week-old seedling roots and leaves, heading-stage flag leaves, young inflorescences (< 2 cm, 2–5 cm, > 5 cm), spikelets (1 day after fertilization [DAF]), and 5 DAF seeds (Fig. 1B and Fig. S2). No expression of *OsCYCD7;1* was seen while *OsCYCD1;1*, *OsCYCD2;3*, and *OsCYCD4;1* were expressed at low levels in all examined tissues. The transcript levels of *OsCYCD* genes were generally not high in parts of the plant associated with cell proliferation, indicating that low doses of *OsCYCD* transcripts were sufficient to drive the G_1_/S transition. It appeared that different *OsCYCD* genes were responsible for proliferation in different cell types. *OsCYCD3;2*, *OsCYCD5;1*, *OsCYCD5;2*, and *OsCYCD5;3* were relatively strongly expressed in the root, where they might play essential roles in cell division in the apical meristem, while *OsCYCD2;2*, *OsCYCD4;2*, *OsCYCD5;2*, and *OsCYCD5;3* levels were high during the early developmental stages of inflorescence which involves meiosis and mitosis during the development of female and male gametophytes. *OsCYCD1;2*, *OsCYCD3;1*, *OsCYCD5;2*, *OsCYCD5;3*, and *OsCYCD6;1* were highly expressed in 1 DAF spikelets which contain active embryogenetic processes while only OsCYCD5;3 was highly expressed during endosperm cell proliferation (5 DAF seeds). The expression levels of OsCYCD5;3 were relatively high in rice, suggesting that this gene might be involved in general mechanisms associated with mitosis and endoreduplication. Although it is accepted that leaf epidermal pavement cells are quiescent as they are terminally differentiated [32], several *OsCYCD* genes (such as *OsCYCD1;2*, *OsCYCD3;2*, and *OsCYCD6;1*) were found to be strongly expressed in both seedling and mature leaves, with levels over 10 times higher than the highest transcript levels of *OsCYCDs* in other tissues. The results suggested that *OsCYCDs* might be also involved in endoreduplication or other processes besides mitosis.

### Analysis of interactions between OsCYCDs and OsRBRs

**Fig. 2:**
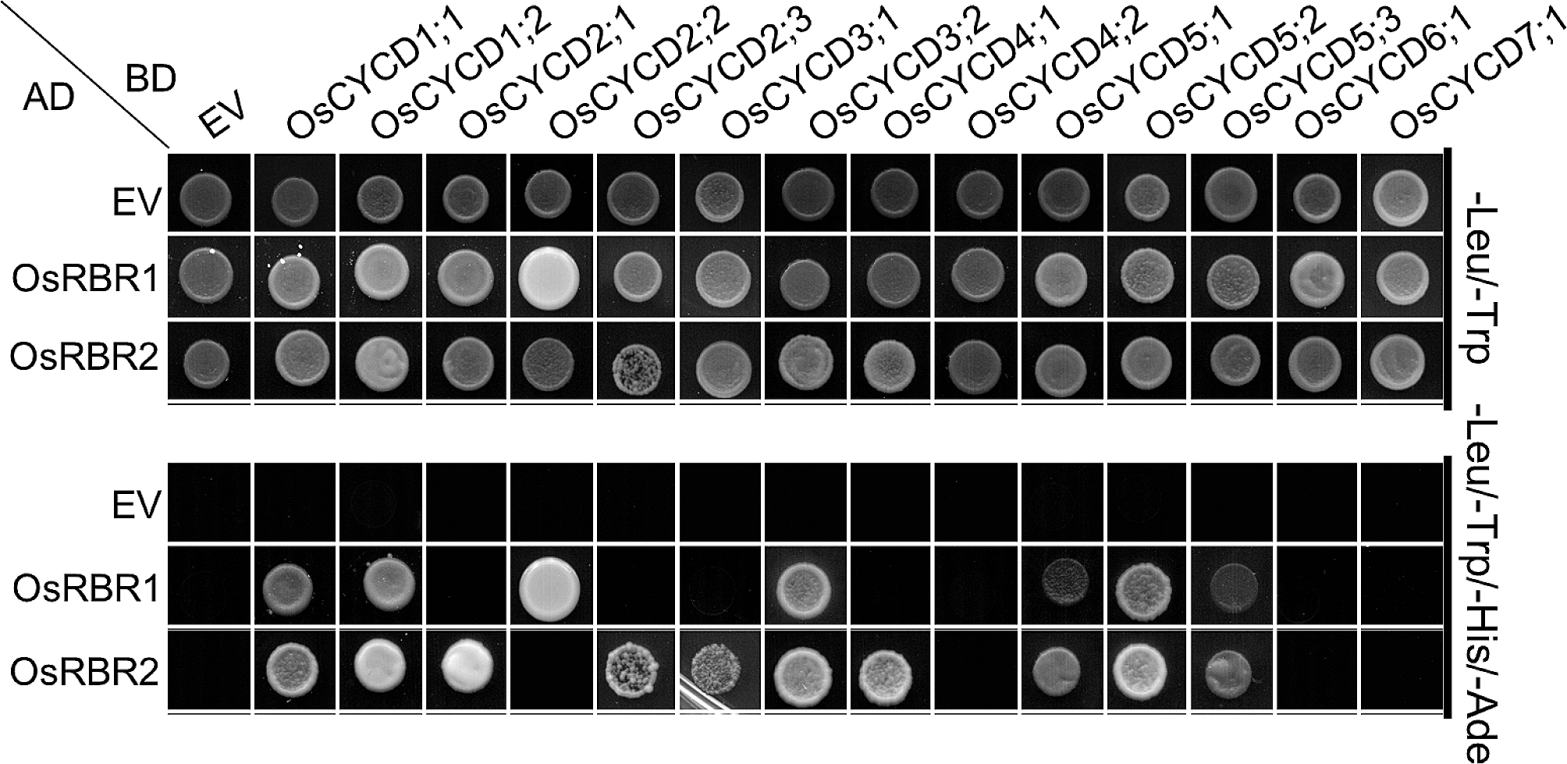
Y2H Assay of the interaction between OsCYCDs and OsRBRs. The up panel indicate the co-transfomed yeast cells spotted onto nonselective medium lacking Leu and Trp, and the lower panel indicate those on selective medium lacking Leu, Trp, His, and Ade

The conserved LxCxE motif at the N-terminus is essential for RBR binding [19]. Two RBR genes, OsRBR1 and OsRBR2, were identified in the rice genome [33]. We used a Yeast two-hybrid (Y2H) system to identify the interaction patterns between OsCYCDs and OsRBRs, OsCYCDs coupled with the DNA-binding domain (BD) and OsRBRs fused to the activation domain (AD). Yeast with co-transformation of OsCYCDs-BD and empty AD vectors showed no growth on SD/-Ade/-His/-Trp/-Leu media, implying that the 14 OsCYCDs had no transcriptional activation activities (Fig. 2). Despite containing the RBR-binding site, OsCYCD4;2, OsCYCD6;1 and OsCYCD7;1 also could not interact with OsRBRs. OsCYCD1;1, OsCYCD1;2, OsCYCD3;2, OsCYCD5;1, OsCYCD5;2, and OsCYCD5;3 could bind to both two OsRBRs proteins while OsCYCD2;1, OsCYCD2;3, OsCYCD3;1, and OsCYCD4;1 only interacted with OsRBR2 and OsCYCD2;2 only bound to OsRBR1 (Fig. 2).

**Fig. 3:**
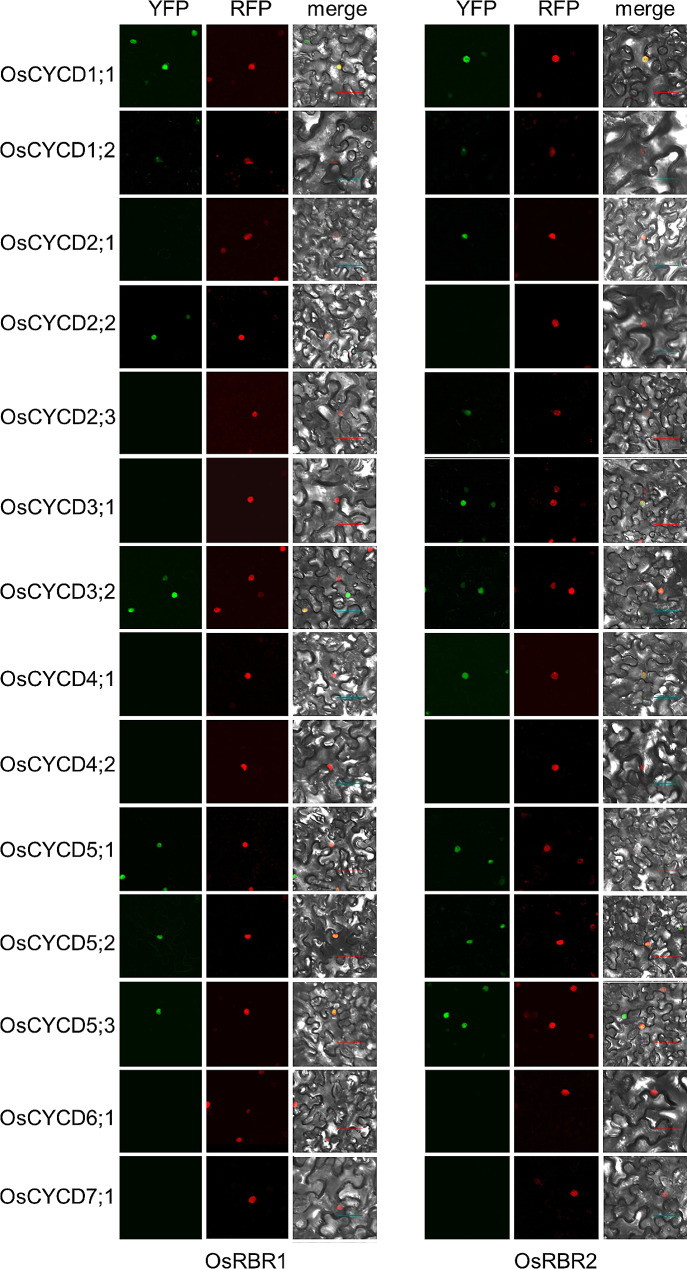
BiFC assay of the interaction between OsCYCDs and OsRBRs in *Nicotiana benthamiana* pavement cells. 14 *OsCYCD* genes were fused to nYFP, and OsRBR1 and OsRBR2 were fused to cYFP. Histone H1.2-RFP is co-transformed and used as a nuclear marker. Bars = 50 μm

Bimolecular Fluorescence Complementation (Bi-FC) assays were employed to examine interactions between the OsCYCDs and OsRBRs in plant cells. The results confirmed those of the Y2H assays. In the abaxial epidermal cells of tobacco leaf, OsCYCD-OsRBR interactions characterized by strong fluorescence of yellow fluorescent protein (YFP) occurred in the nucleus labeled by AtHistone H1.2-TagRFP in epidermal cells of co-infiltrated *Nicotiana benthamiana* leaves. In particular, OsCYCD1;2 interacted with all OsRBR proteins in the nucleus or nucleolus; on the contrary, the lack of an obvious YFP signal in cells with a TagRFP signal indicated that there was no interaction between the corresponding OsCYCDs and OsRBRs (Fig. 3). In additional, any co-transformation of p35S::OsCYCD-nYFP plasmids and empty p35S::cYFP vector, or empty p35S::nYFP vector and p35S::OsRBR-cYFP plasmids did not produce YFP fluorescence (Fig. S3 and Fig. S4). These results suggest that most OsCYCDs (except OsCYCD4;2, OsCYCD6;1, and OsCYCD7;1) physically interact with at least one OsRBR protein both in vitro and in vivo, and their interaction properties are not the same.

### Analysis of interaction between OsCYCDs and OsCDKAs

**Fig. 4:**
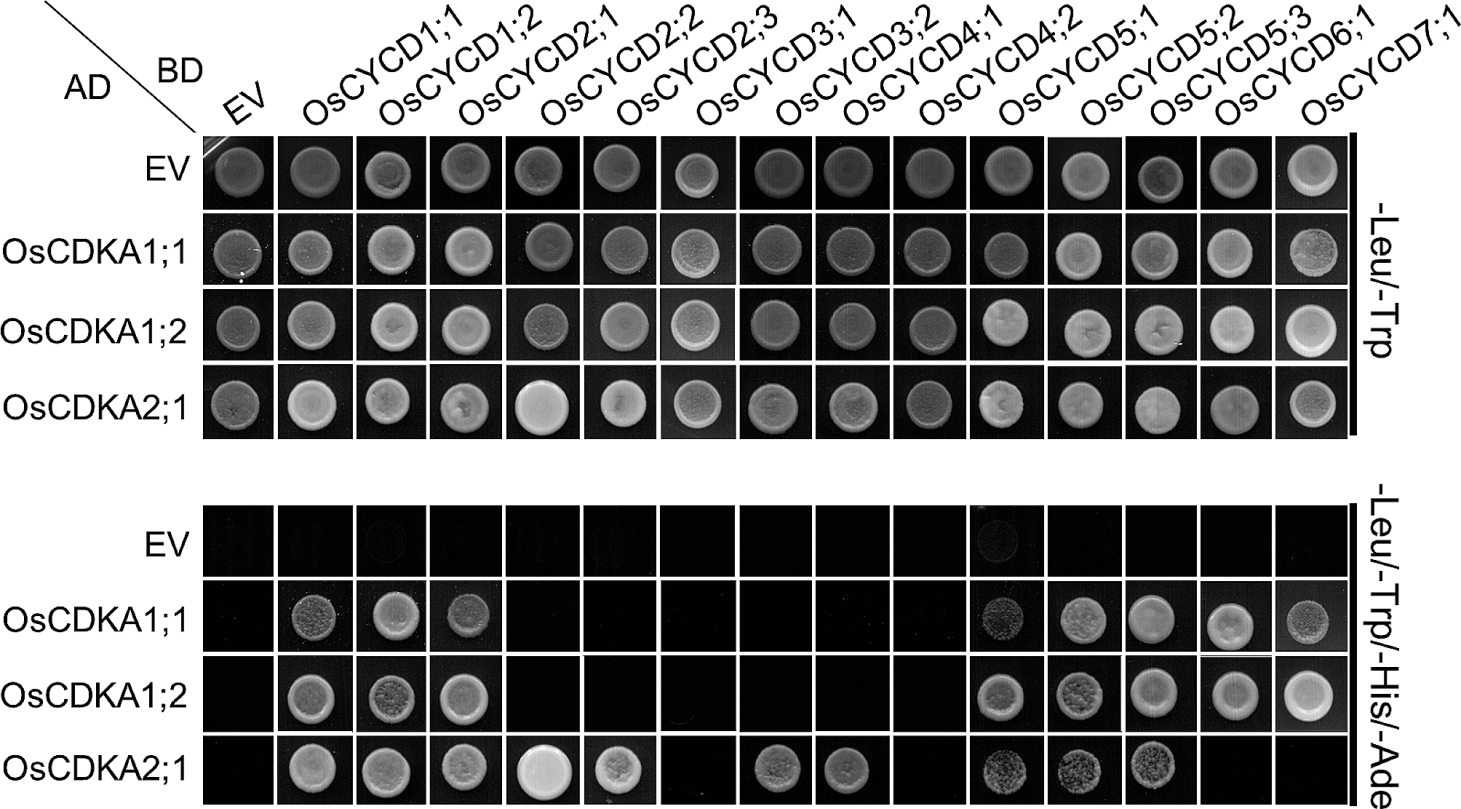
Y2H Assay of the interaction between OsCYCDs and OsCDKAs. The up panel indicate the co-transfomed yeast cells spotted onto nonselective medium lacking Leu and Trp, and the lower panel indicate those on selective medium lacking Leu, Trp, His, and Ade

**Fig. 5:**
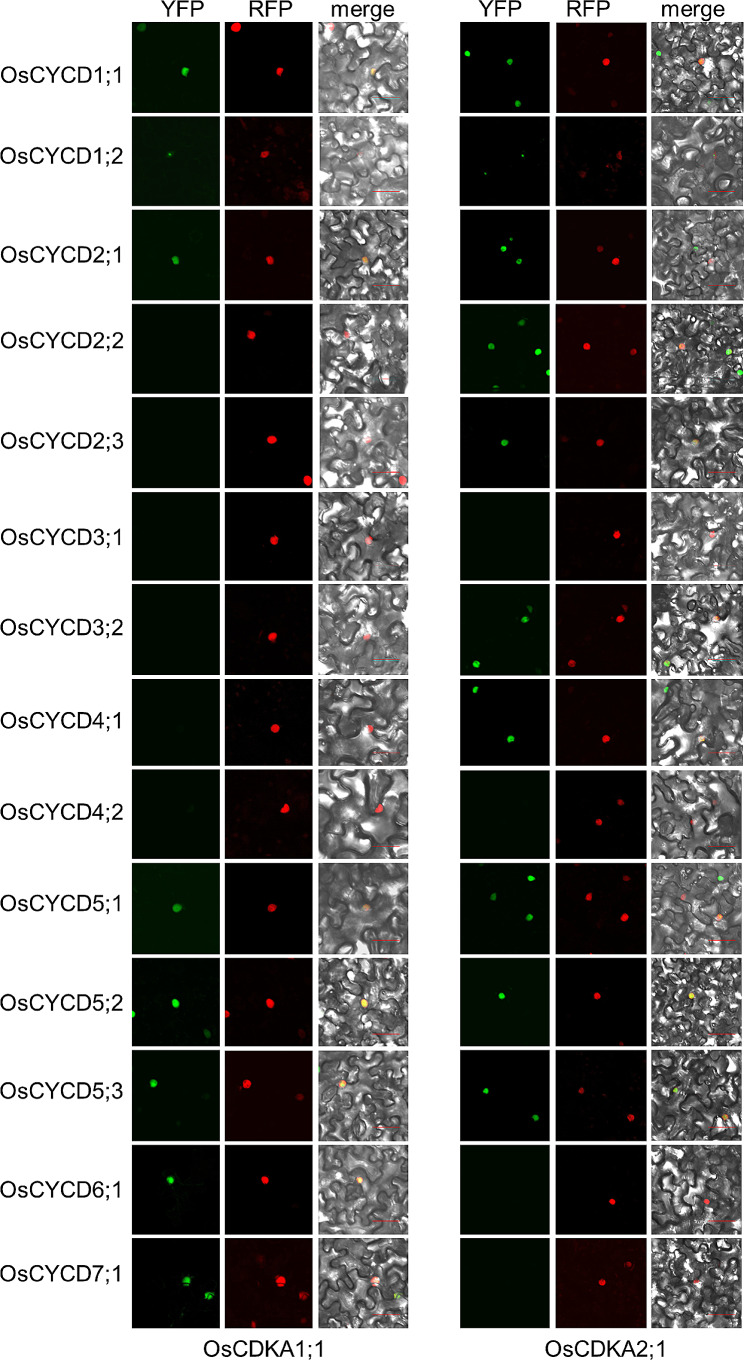
BiFC assay of the interaction between OsCYCDs and OsCDKAs in *Nicotiana benthamiana* pavement cells. 14 *OsCYCD* genes were fused to nYFP, and OsCDKAs were fused to cYFP. Histone H1.2-RFP is co-transformed and used as a nuclear marker. Bars = 50 μm

All CYCDs contain cyclin N domains which are essential for the interaction between the cyclin and its CDK binding partner (CDKA in plants) [34]. Although all 14 CYCDs in rice contain the cyclin signature, it was still necessary to determine their interactions with OsCDKAs. Three OsCDKA proteins were identified in the rice genome, including LOC_Os03g02680, LOC_Os03g01850, and LOC_Os02g03060 which were termed OsCDKA1;1, OsCDKA1;2 and OsCDKA2;1. The interactions between OsCYCDs and OsCDKAs were then investigated using both Y2H and Bi-FC, leading to consistent results (Figs. 4 and 5, and Fig. S5). OsCDKA1;1 and OsCDKA1;2 show strong similarity (up to 96%), and showed the same interaction patterns with OsCYCDs. Seven OsCYCDs (including OsCYCD1;1, OsCYCD1;2, OsCYCD2;1, OsCYCD2;2, OsCYCD5;1, OsCYCD5;2, and OsCYCD5;3) were found to physically interact with either OsCDKA1 or OsCDKA2 while five OsCYCDs interacted with only one subfamily of OsCDKAs, with OsCYCD6;1, and OsCYCD7;1 binding to OsCDKA1, while OsCYCD2;3, OsCYCD3;2, and OsCYCD4;1 were observed to bind to OsCDKA2; Although harboring the cyclin signature, OsCYCD3;1 and OsCYCD4;2 were unable to interact with either OsCDKA1 or OsCDKA2 (Fig. 4). In tobacco leaf pavement cells, it was found that all the positive OsCYCD-OsCDKA interactions occurred in the nucleus, although the OsCYCD1;2-OsCDKA interaction might occur in the nucleolus; on the contrary, the lack of an obvious YFP signal in cells with a TagRFP signal indicated that no interaction occurred between the OsCYCDs and OsCDKAs (Fig. 5 and Fig. S5), and no YFP fluorescence signals were observed in the controlled experiments (Fig. S3 and Fig. S4). These results suggest that most OsCYCDs (except OsCYCD3;1 and OsCYCD4;2) physically interact with at least one OsCDKA protein both in vitro and in vivo.

### Mitotic cell division in tobacco leaf cells after ectopic OsCYCD expression

**Fig. 6:**
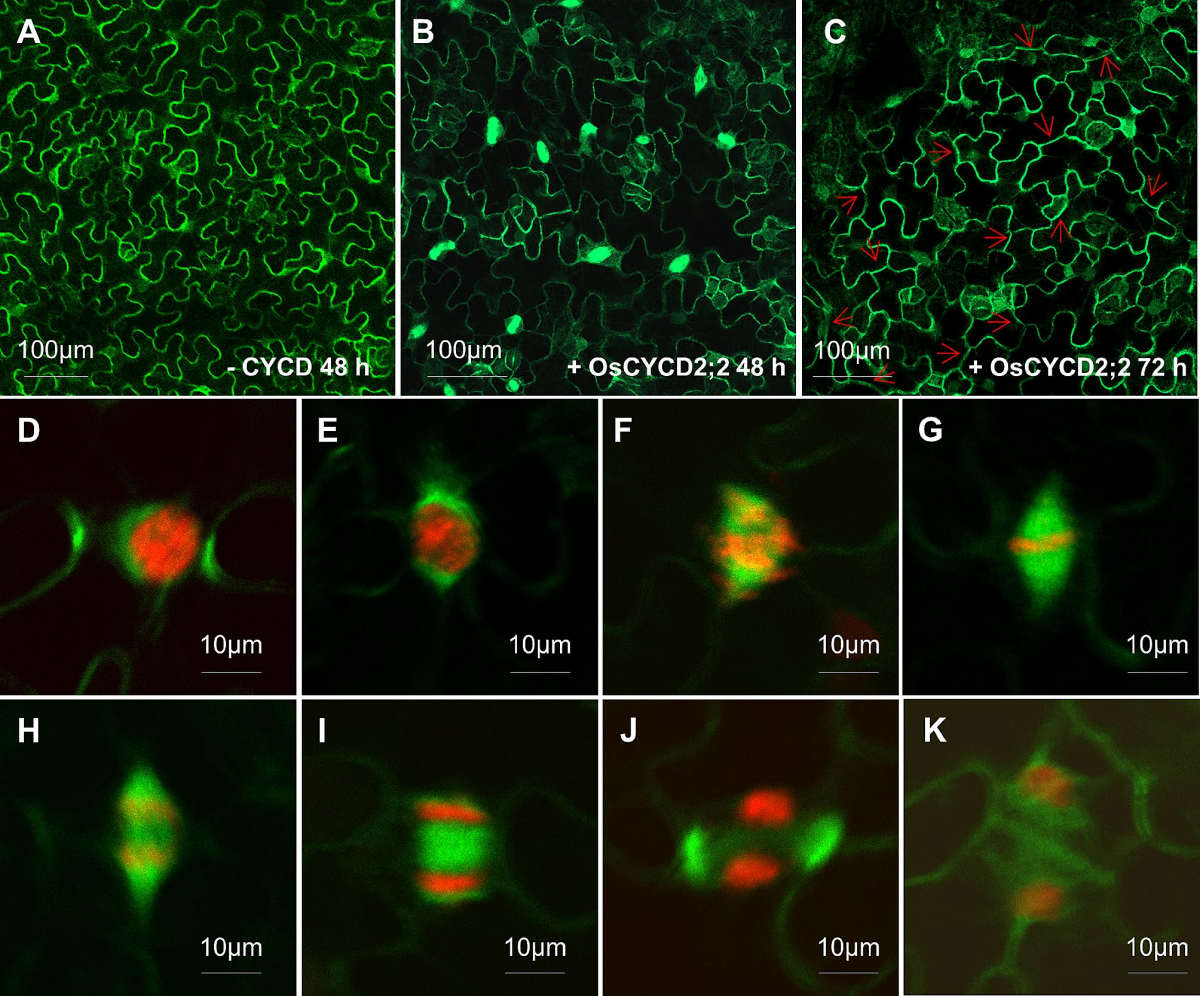
Transient expression of *OsCYCDs* triggers tobacco pavement cells to divide. **(A)** Differentiated leaf pavement cells are outlined by microtubules at the cell cortex without ectopic expression of any OsCYCDs. **(B)** Upon *OsCYCD2;2* expression, leaf pavement cells enter mitotic cell division, obvious mitotic signs such as spindles and phragmoplasts were observed in green. **(C)** Seventy-two hours after infiltration of agrobacteria with *OsCYCD2;2*, most if not all pavement cells have already completed cell division as marked by cell plates (red arrows). An induced pavement cell forms a preprophase microtubule band **(D)**, and cells in early-prophase **(E)**, propahse **(F)**, metaphase **(G)**, anaphase **(H)**, early-cytokinesis **(I)**, late-cytokinesis **(J)**, and completed division **(K)**. Microtubules are marked by GFP-TUA6 (green) and nuclei highlighted by AtHistone H1.2-TagRFP (red). Bars: (**A**–**C**) 100 μm; (**D**–**K**) 10 μm

Based on above results, not all OsCYCDs could interact with both CDKA and RBR to format the mitosis triggering module (CYCD-CDKA-RBR complex), it is important to analyze their roles in cell division. Our previous work demonstrated that transient expression of *AtCYCD3;1* and *AtCYCD4;2* induced differentiated leaf cell entry to cell division, which named Cell Division-Enabled Leaf System (CDELS) [21]. Here, we used the same system to test the effects of rice OsCYCDs on mitotic cell division. The fully expanded pavement cells expressing any of the OsCYCD proteins (except OsCYCD7;1) showed the presence of spindle and phragmoplast microtubule (MT) arrays (Fig. 6B). It was further observed that at 72 h following infiltration, the majority of pavement cells showed completion of CYCD-induced cell division, resulting in the formation of mature cell plates (Fig. 6C). When ectopic expression of *OsCYCDs* was induced, the pavement cells re-entered mitosis, completing all phases. The spindle and phragmoplast MT arrays together with chromosomal configurations (labeled by AtHistone H1.2-TagRFP during transient expression), allowed the easy observation of the MT pre-prophase band (Fig. 6D), MT polymerization on the nuclear envelope during late prophase (Fig. 6E–F), chromosomal alignment at the metaphase plate (Fig. 6G), and separation at anaphase (Fig. 6H). The formation of daughter nuclei and new cell plates at the neck of the pavement cells (Fig. 6I–J), leading to reduced cell plate surface area (Fig. 6K), could also be observed. In contrast, lobed pavement cells lacking *OsCYCD* expression were unable to enter cell division (Fig. 6A). These results revealed that all OsCYCDs except OsCYCD7;1 stimulated tobacco pavement cell re-entry into mitosis, regardless of their ability to interact with CDKA or RBR proteins.

### Different effects of ectopic OsCYCDs expression on mitotic cell division

**Fig. 7:**
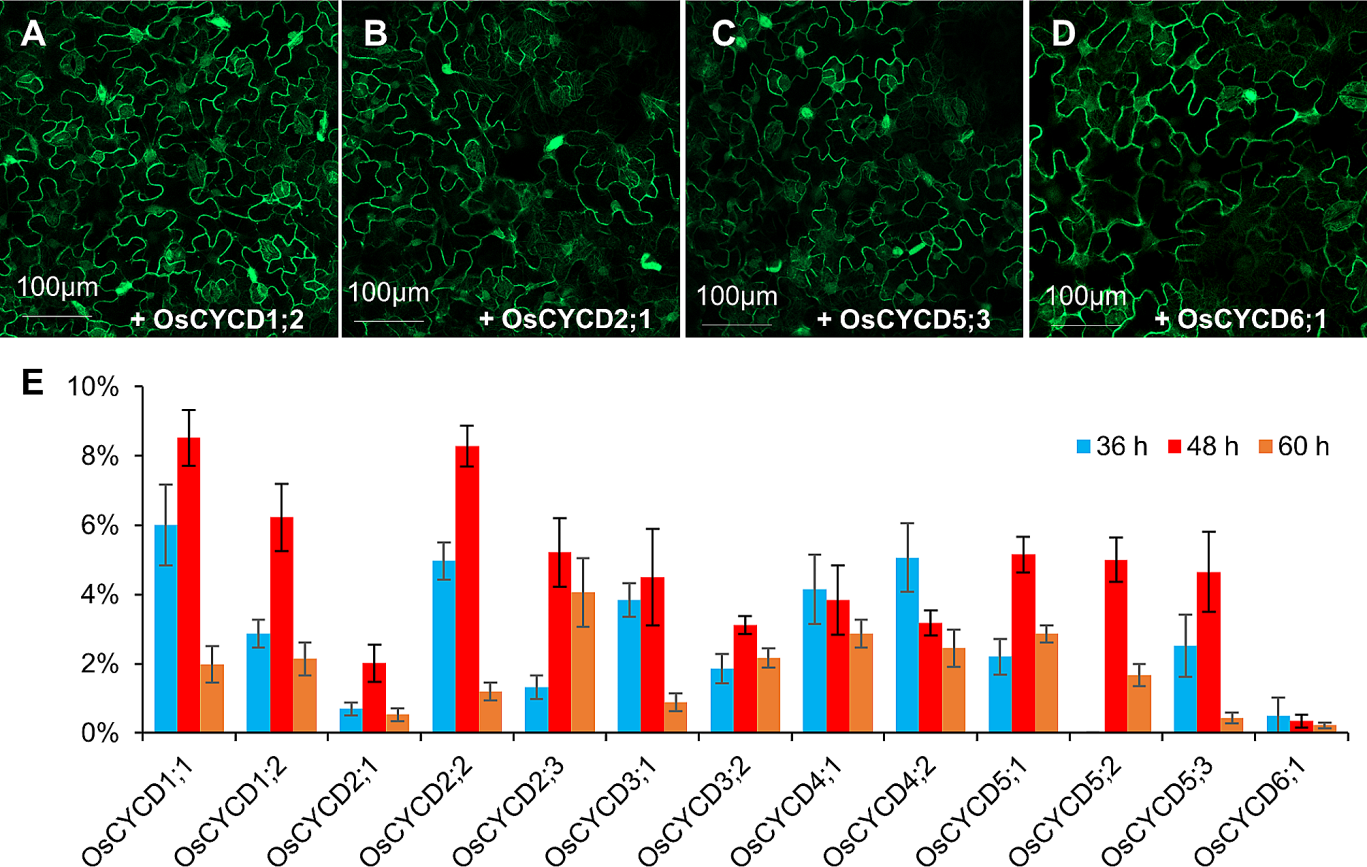
Frequencies of pavement cells at mitosis upon the expression of different *OsCYCD* genes. Tobacco leaf pavement cells enter mitotic cell division upon expression of *OsCYCD1;2* **(A)**, *OsCYCD2;1* **(B)**, *OsCYCD5;3* **(C)**, and *OsCYCD6;1* **(D)**. **(E)** Frequencies of pavement cells at mitosis. Samples were examined at 36 h, 48 h, and 60 h after agrobacterial infiltration. Twelve independent samples were examined at each time point and 300 cells were counted for each sample. Bars = 100 μm

Although 13 OsCYCDs were found to induce tobacco leaf pavement cell re-entry to cell division, additional cells undergoing mitosis were easily observed in some leaves expressing *OsCYCD* genes, while significantly fewer cells were observed to undergo mitosis after the expression of other genes. We then analyzed the percentages of epidermal cells undergoing mitotic cell division after infiltration to examine the effects of different OsCYCDs. There were significantly more cells with spindles or phragmoplasts in leaves injected with OsCYCD1;2 (Fig. 7A) and OsCYCD5;3 (Fig. 7B) than those injected with OsCYC2;1 (Fig. 7C) and OsCYCD6;1 (Fig. 7D). Very few mitotic cells were observed 24 h after infiltration with any OsCYCD (data not shown), while numerous cells with mitotic MT arrays were observed 36 or 48 h after infiltration, after which the count decreased at 60 h (Fig. 7E). The numbers of mitotic cells expressing *OsCYCD4;1*, *OsCYCD4;2*, and *OsCYCD6;1* peaked at an earlier time (36 h after infiltration), while the numbers of cells with other *OsCYCD* genes peaked 48 h after infiltration. Most of the genes had similar effects, with 4–6% of cells undergoing division at the peak time after the expression of *OsCYCD1;2*, *OsCYCD2;3*, *OsCYCD3;1*, *OsCYCD4;1*, *OsCYCD4;2*, *OsCYCD5;1*, *OsCYCD5;2*, and *OsCYCD5;3*, while the peak values after *OsCYCD2;1*, *OsCYCD3;2*, and *OsCYCD6;1* infiltration were less than 4%, especially for *OsCYCD6;1* where the frequency was about 1%. OsCYCD1;1 and OsCYCD2;2 were found to be the most efficient promoters of the cell cycle, with peak values of about 8%. These results suggest that D-type cyclins in rice have relatively conservative functions in activating cell division, while variations in their sequences result in differences in efficiency.

### OsCYCDs regulate cell division in rice

**Fig. 8:**
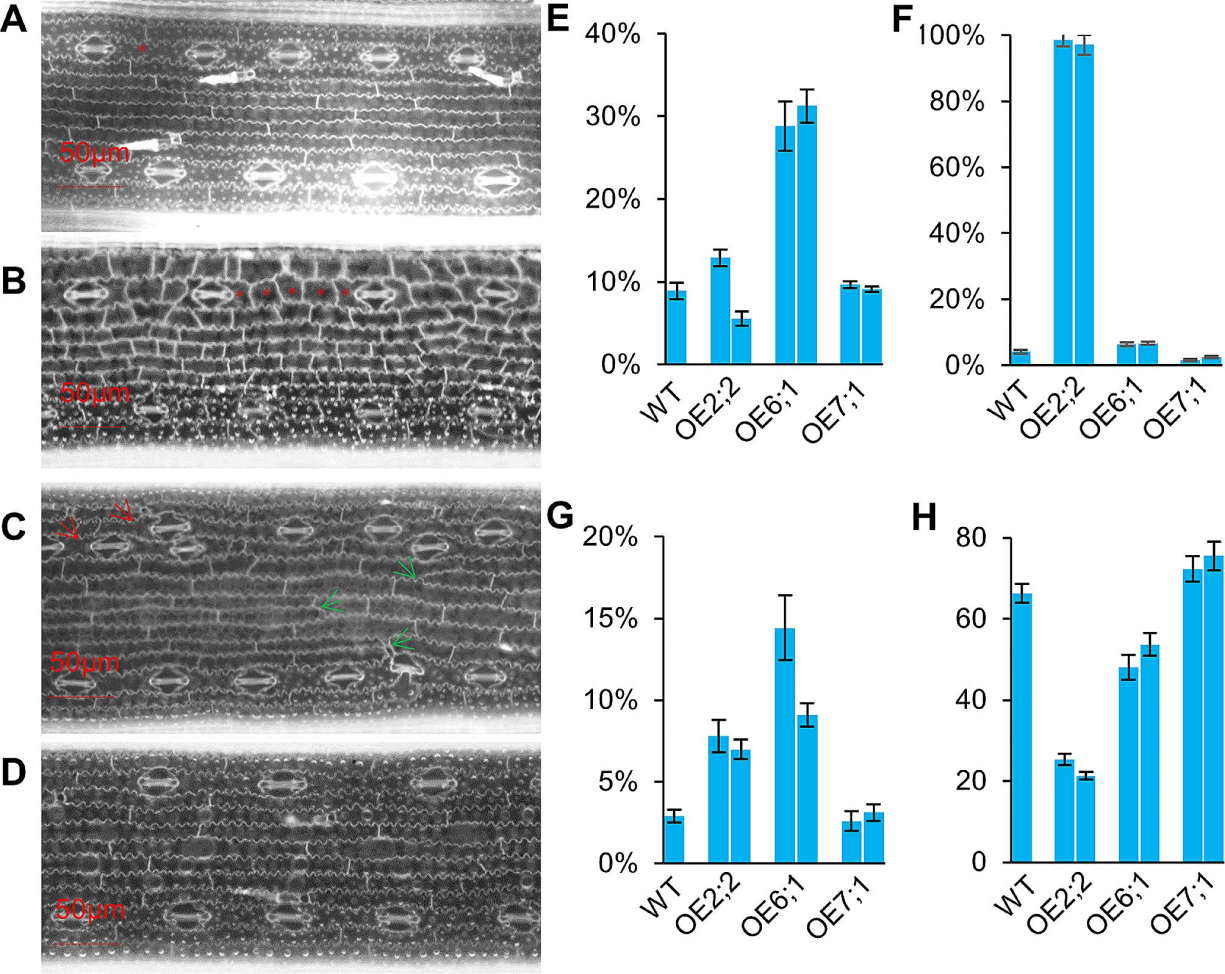
Cell division in leaves of *OsCYCDs* overexpressing plants. The arrangements of cells in both stomata files and the non-stomata area in WT **(A)**, *OsCYCD2;2* ovexpressing plants **(B)**, *OsCYCD6;1* ovexpressing plants **(C)**, and *OsCYCD7;1* ovexpressing plants **(D)**. Red stars represent single cell or tandem multiple cells between two stomata. Red arrows represent two neibouring files containing stomata (somatal file twins, SFT) which might be caused by the abnormal proliferation of stomatal-lineage cells. Green arrows represent additional files between two stomata files supererogatory divisions occurred at partial epidermal cells. **(E)** The rate of SFT in WT and *OsCYCDs* overexpressing plants. **(F)** The rate of single cell and tandem multiple cells between two stomata in WT and *OsCYCDs* overexpressing plants. **(G)** The rate of additional files between two stomata files in WT and *OsCYCDs* overexpressing plants. **(H)** The length of cells in the non-stomata area. Each line were examined with five independent samples. Bars = 50 μm

To elucidate the functions of OsCYCDs in the rice cell cycle, *OsCYCD2;2*, *OsCYCD6;1*, and *OsCYCD7;1* (which induced re-entry of pavement cells into cell division with high-, low-, and zero-efficiencies, respectively) were over-expressed in rice. Quantitative real-time PCR (qRT-PCR) revealed that their transcript levels were increased by at least 10 times relative to the levels in wild-type seedlings (Fig. S6). The leaves of WT and transgenic plants were stained with propidium iodide, and cell sizes and cell counts were analyzed by the elaboration of lobes outlined by red fluorescent signals. The morphology of stomata was observed to be normal in all plants, being formed from dumbbell-shaped guard cells flanked by subsidiary cells (Fig. 8A–D), suggesting that these OsCYCDs did not affect asymmetric and symmetric divisions during the development of rice stomatal. During stomatal development, potential precursors of stomatal-lineage cells proliferate in specific files, after which cells enter asymmetric and symmetric divisions to form the stomata [35]. In WT leaves, most stomatal files were observed to lack neighbors, with only 8.9% stomatal file twins (SFT) and two neighboring files containing stomata observed, which might be caused by the abnormal proliferation of stomatal-lineage cells (Fig. 8A, E). The SFT rates in OE-*OsCYCD2;2* and OE-*OsCYCD7;1* plants did not change significantly (Fig. 8B, D–E), and in OE-*OsCYCD6;1* leaves, about 30% of stomatal files were found to be SFT (Fig. 8C, E). In the stomatal files, according to the pathway of stomatal development, there is usually only one normal cell between two stomata, with multiple cells rarely observed; low numbers of double cells in the stomatal intervals were seen in WT, OE-*OsCYCD6;1*, and OE-*OsCYCD7;1* leaves (4.1%, 6.5%, and 2.5%, respectively). However, there were few observations of single cells in the stomatal intervals in OE-*OsCYCD2;2* leaves while a large proportion of multiple cells between two stomata was seen, including 24.2% two-cells, 34.8% three-cells, 25.8% four-cells, 12.1% five-cells, and 1.5% six-cells (Fig. 8A–D, F).

In the non-stomatal area, the epidermal cells were neatly arranged between two stomata files caused by disciplinary cell division. Compared with the WT, more supererogatory divisions occurred in partial epidermal cells leading to additional files in OE-*OsCYCD2;2* and OE-*OsCYCD6;1* (Fig. 8C, G), while the percentage of additional files in OE-*OsCYCD7;1* was similar to that in the WT. The cell counts and lengths were analyzed (Fig. 8H). The length of cells in the non-stomatal areas of OE-*OsCYCD2;2* and OE-*OsCYCD6;1* leaves were significantly less than those in the WT, especially in the former, where the length was only one-third that of the WT. On the contrary, the cells in OE-*OsCYCD7;1* were slightly longer than those in the WT (Fig. 8A–D, H). Therefore, in leaves of the same length, there were more cells in OE-*OsCYCD2;2* and OE-*OsCYCD6;1* plants than in the WT, while the least cells were observed in OE-*OsCYCD7;1* plants. Taken together, overexpression of *OsCYCD2;2* and *OsCYCD6;1* promoted division of both stomatal lineage and epidermal cells, with *OsCYCD2;2* appearing to be more effective; in contrast, overexpression of *OsCYCD7;1* was not only ineffective, but even inhibited cell division. The efficiencies of inducing cell division by OsCYCD2;2, OsCYCD6;1, and OsCYCD7;1 in rice leaves (Fig. 8) were consistent with those observed in CDELS (Fig. 7).

## Discussion

### OsCYCDs are involved in cell proliferation in different tissues

Overall, the CYCD family shows varied expression patterns in rice (Fig. 1B). Most of the genes were expressed in all the tissues investigated, although expression varied according to tissue. *OsCYCD5;3* was relatively strongly expressed in all tissues, while *OsCYCD1;1*, *OsCYCD3;3*, and *OsCYCD4;1* were expressed at lower levels, and *OsCYCD7;1* was rarely detected; other *OsCYCDs* were highly expressed in one or more specific tissues. The functions of OsCYCD3;2 were identified, finding that knockout of the gene reduced the activity of the axillary meristem and SAM, resulting in significantly fewer branches [30]. *OsCYCD3;2* was found to be highly expressed during the young inflorescence stage (0–2 cm), associated with branch formation. These findings indicate that some CYCDs might be involved in the basic process of cell division, while others may play essential roles in specific tissues.

The hypothesis has been verified by the functions of CYCDs in *Arabidopsis*. There are 10 CYCD proteins in *Arabidopsis*, with overexpression of most of these genes observed to promote the division of various cell types [24, 25, 27]. However, the loss-of-function mutants of CYCDs show various morphological features, suggesting that these genes affect cell division according to their specific expression patterns. *AtCYCD2;1* is expressed in the nuclei of root cells, especially in apical and lateral meristems. Knockout of *AtCYCD2;1* has minimal influence on the densities of lateral roots [36]. The three *AtCYCD3* genes are expressed specifically in the cambium during vascular development, with the triple knockout showing markedly smaller diameters resulting from reduced mitosis in the stem and hypocotyl cambium [37]. *AtCYCD4;1* has been observed to be expressed in a variety of tissues, while *AtCYCD4;2* was not found in shoot and root meristems [27]. Although mutants of both *AtCYCD4* genes showed normal growth with no phenotypic alterations, the *atcycd4;1* mutant led to a displaced apical/basal meristem boundary in the pericycle together with increased lengths of the basal meristem cells [38]. A later study showed that knockout of *AtCYCD4;1* reduced the speed of seed germination [39]. Significantly reduced stomatal numbers were seen in both *atcycd4;1* and *atcycd4;2* while no marked changes in the epidermal cells were observed [27]. *AtCYCD5;1* was shown to be a QTL controlling endoploidy by modulation of endocycle progression, and *CYCD5;1* knockout resulted in reduced DNA ploidy levels [40]. Endopolyploidy is commonly seen in seed plants and is caused mostly endoreduplication where additional rounds of genomic duplications occur in the absence of mitosis [41]. *OsCYCD5;3* a homolog of *AtCYCD5;1*, was found to be strongly expressed in all tissues, indicating that it may serve in a common cell-cycle pathway, possibly endoreduplication. *AtCYCD7;1* expression occurs immediately before the division of symmetric guard cells, where it acts to limit division [29]. *OsCYCD7;1* was hardly detected in any tissues, suggesting the possibility of highly specific functions; moreover, overexpression of *OsCYCD7;1* did not stimulate tobacco pavement cell re-entry into cell division and reduced cell division in rice leaves (Figs. 7 and 8).

Analysis of tissue-specific expression represents only the initial step in understanding the functions of *CYCD* genes. Many *CYCD* genes were expressed differently in different tissues, providing clues for further functional analysis. For example, several *OsCYCDs* expressed at higher level in the mature leaf than in the young proliferating one, suggesting they might positively regulate cellular growth which was similar to a single CYCD homolog in Drosophila [42]. Detailed morphological and cytological analyses, together with overexpression and knockout studies, will clarify the functions of these genes in rice.

### The functions of OsCYCDs might not completely depend on the CDKA-CYCD-RBR complex

It is well-known that CYCDs interact with CDKAs and RBRs and regulate the activity of E2F/DP [12, 13, 43]. Although all 14 CYCD proteins in rice contained the cyclin signature, CDK phosphorylation sites, and LxCxE motif which is responsible for CDKA and RBR binding, only 12 of them were found to interact with at least one OsCDKA protein and 11 CYCDs interact with at least one OsRBR. Taken together, only 10 OsCYCDs were capable of forming the entire CYCD-CDKA-RBR complex; OsCYCD3;1, OsCYCD6;1, and OsCYCD7;1 most likely to interact with only one of two other components, while OsCYCD4;2 possibly bound neither CDKAs nor RBRs. However, all rice CYCD genes except OsCYCD7;1, were able to induce ectopic cell divisions in tobacco; moreover, both *OsCYCD2;2* and *OsCYCD6;1* overexpression promoted cell division in rice leaves. These results suggested that CYCDs might regulate the cell cycle through some novel pathways besides CDKA-CYCD-RBR manner.

Both plants and animals have different CDKs leading to the formation of various CDK-cyclin complexes controlling distinct aspects of the cell cycle [22, 44–46]. Plant CDKs are classified into eight types according to their protein sequences, namely, the CDKA–CDKG and CDK-like kinases [47, 48]. In animals, CDK1 (Cdc2) promotes entry into mitosis while, in plants, both A-type (CDKA) and B-type (CDKB) CDKs are involved [45, 46, 49]. D-type cyclins usually bind to CDKA while cyclins A and B bind CDKB [34, 50]; for example, AtCYCD3;1 binds CDKA but not CDKB, to form an active complex [34]. CYCDs may also bind to other CDKs. CDKB2;1 can interact with both CYCD1;1 and CYCD4;1 [51], while D3 cyclins in tobacco (3;3 and 3;5) bind CDKA and CDKB in vitro and, following activation, phosphorylate histone H1 and NtRBR1 [52]. In maize, CYCD2;2, CYCD4;2, and CYCD5;3 interact with CDKA and CDKB, resulting in various patterns of binding and activation during germination [53]. Maize CycD3;1 also interacts with CDKA and CDKB1;1 and uses histone H1 to acquire kinase activity [54]. In poplar, CYCD3;1 and CYCD3;3 bound to both CDC2 and CDKE;1, while the D5 proteins interacted with CDKA, CDKB1;2, CDKD1;1, CDKD1;3, CDKE;1, and CDKF, and D6 proteins bound CDKA, CDKB1;2, CDKD1;1, and CDKD1;3 [55]. Furthermore, Cruz-Ramínez group indicated that Arabidopsis CYCD6;1 interacted with RBR in Arabidopsis protoplasts; CYCD6;1 also interacted with CDKB1 and formed an active complex that phosphorylates RBR [56]. These findings suggest that rice CYCD proteins may associate with different CDKs to form active complexes for RBR phosphorylation and the cell cycle activation.

Unlike CDKA, no RBR homologs have been identified. However, it has been found that AtCYCD4;2, an atypical D-type cyclin that does not contain the Rb-binding motif but can still promote cell division [57], could rescue G_1_ in cyclin-deficient yeast, with overexpression leading to more rapid callus formation in hypocotyl explants in comparison with the wild-type [57]. Markedly reduced numbers of stomata were seen in *atcycd4;2* mutants, with the opposite effects observed in CYCD4;2-OE hypocotyls [27]. Our earlier study showed that ectopic expression of *AtCYCD4;2* promoted re-entry of cell division in tobacco pavement cells [21]. These findings suggest the likelihood of Rb/E2F/DP pathway-independent stimulation of cell division in plants. It is possible that CYCD-CDKs could phosphorylate substrate(s) other than Rb, potentially leading to cell division. CYCDs may also bind to specific transcription factors or hormone receptors, as has been shown in mammals where direct associations with transcription factors (such as DMP1, C/EBPß/Nf-I16, and AML1) and androgen and estrogen receptors have been observed, altering transcriptional activation independently of CDKs [58–62]. However, there are no reports of such mechanisms in plants, suggesting the possibility of future investigations.

## Conclusions

In this study, we identified 14 D-type cyclins in the rice genome. These CYCD proteins were classified into seven subtypes based on their evolutionary relationships. The genes showed distinctive tissue expression patterns, suggesting a diversity of functions in controlling the proliferation of different cell types. The CYCDs might have different interaction patterns with both CDKs and RBRs, with most, apart from OsCYCD7;1, inducing cell division, although the effects on cell division varied. These findings expand our understanding of plant CYCDs and their involvement in cell division and lay a solid foundation for future functional studies on the CYCDs of rice.

## Materials and methods

### Database searches, identification of domains, and phylogenetic analysis

Rice CYCD proteins were identified in two Rice Annotation Project Database Web sites (http://rapdb.dna.affrc.go.jp/ and http://rice.uga.edu/index.shtml) using BLASTP with *Arabidopsis* CYCD proteins and annotations. Maize CYCD proteins identified by PsiBLAST against the NCBI databases. Conserved domains and motifs were identified using MEME. ClustalW was used for the alignment of the full protein sequences. Phylogenetic trees were constructed using the maximum likelihood method, with 1000 bootstrap values presented at the nodes of the unrooted trees.

### Plant materials and growth conditions

The cultivar “Nipponbare (*Oryza sativa* L. spp. *japonica*)” was used for the experiments. The seeds were germinated and cultured to three-leaf seedlings in a growth chamber at 30℃/25℃ with a day/night light cycle. The seedlings were subsequently transplanted to a paddy field at Jiangxi Agricultural University in Nanchang (28.77ºE, 115.84ºN), Jiangxi Province, China. Both rice seedlings and mature plants were used for the extraction of genomic DNA and total RNA. RNA was extracted from the young leaves and roots of three-week-old seedlings and the mature leaves and panicles of plants at the heading stage.

### RNA extraction and quantitative real-time PCR (qRT-PCR)

Total RNA was extracted using a Plant MiniBEST RNA Extraction Kit (Takara, Japan) and was reverse-transcribed to cDNA using PrimeScript II reverse transcriptase (Takara) according to the provided directions. Triplicate qRT-PCR amplifications per sample were performed using 2×SYBR Green PCR Master Mix (Applied Biosystems, USA) in a 7500 Real-Time PCR System (Applied Biosystems). The relative mRNA expression levels of all rice *CYCD* genes were determined using the 2^−ΔCt^ method, which were normalized to that of the internal control ubiquitin gene. Three biological replicates were used for all experiments. The primers used are listed in Table S1 .

### Plasmid construction and plant transformation

Plasmids were constructed using a recombinase system (Vazyme, China) with direct recombination of the amplified fragments and linearized destination vectors with Exnase II (Vazyme). The PCR templates used for cloning the *CYCD*, *RBR*, and *CDK* genes were a pool of different cDNA preparations from various rice tissues. Amplification was performed with KOD-Fx DNA polymerase (Toyobo, Japan). For *CYCD* gene knockout, gene-editing plasmids were constructed from VK005-09 (Beijing Viewsolid Biotech, China), following the provided directions. The primers used for vector construction are listed in Table S2, Table S3, and Table S4.

Plasmids were transformed into *Agrobacterium tumefaciens* strain GV3101 before infiltration into tobacco plant leaves and strain EHA105 for transformation. Both procedures were performed using previously described protocols [21, 63].

### Yeast two-hybrid (Y2H) assays

Full-length *CYCD* genes were inserted into pGBKT7, and *RBR* and *CDK* genes were inserted into pGADT7. Table S2 lists the primers used. Varying combinations were then co-transformed into AH109 cells for analysis of transactivation. The negative control was pGBKT7/pGADT7 while the positive control was BD-OsTub1/AD-D1. The yeast clones were cultured on synthetic defined (SD) medium without leucine and tryptophan (SD/-Trp/-Leu) for 3–4 days at 30 ℃. Interaction tests were conducted in SD medium without adenine, histidine, Leu, and Trp (SD/-Ade/-His/-Trp/-Leu) with the addition of 5 mM 3-AT (3-amino-1,2,4-triazole). Colony PCR was used for assessing the presence of plasmids in positive clones.

### Bimolecular fluorescence complementation (Bi-FC) assays

The *OsCYCD* cDNAs were individually cloned into p35S::nYFP plasmids, and the *OsRBR* and *OsCDK* genes into p35S::cYFP vectors. Table S3 shows the primers used. *Nicotiana benthamiana* pavement cells were used for co-expression of the recombinant NYFP and CYFP vectors using infiltration by Agrobacterium. Samples of leaves were collected after 48 and 72 h and examined under an Axio Observer inverted microscope equipped with the FV3000 laser scanning confocal module (Olympus). The co-transformation of p35S::OsCYCD-nYFP plasmids and empty p35S::cYFP vector, empty p35S::nYFP vector and p35S::OsRBR(or OsCDKA)-cYFP were used as negative controls.

### Cell division-enabled Leaf System (CDELS) Assays

GV3101 agrobacteria were transformed by plasmids expressing *OsCYCDs*, P19 viral protein, histone H1.2-RFP, respectively. Three types of agrobacteria were mixed and delivered into the GFP-α-TUA6 tobacco leaves. After infiltration (36–72 h), the arrays of spindle and phragmoplast MTs together with their respective chromosomal configurations were examined under confocal microscopy using GFP and TaqRFP standard settings. The numbers of epidermal cells showing evidence of mitosis were determined. Detailed procedures are described in our earlier publications [21].

### Propidium iodide staining

The leaf samples were harvested from three-leaf seedlings. The detached leaves were cut into 2–3 cm pieces and were soaked in staining solution (5 µg/mL propidium iodide in 100 mM phosphate buffer, pH 7.4 ) for 24 h in the dark. The samples were then examined using an Eclipse Ni-U microscope equipped with MRD71670 and MRD71970 CFI Plan Apochromat Lambda D objectives (Nikon), imaged with a Panda sCMOS camera (PCO Imaging), and the images were analyzed using Nikon software. The size, length, and width of cells were analyzed in EIS-Elements D 5.3 software.

### Electronic supplementary material

Below is the link to the electronic supplementary material.

Supplementary Material 1

## Data Availability

No new sequence was generated in the current study. All data generated or analysed during this study are included in this published article and its supplementary information files.

## Abbreviations

CYCD: Type cyclin
RBR: Retinoblastoma related protein
CDKA: A-type cyclin-dependent kinases
Y2H: Yeast two hybrid
BD: DNA-binding domain
AD: Activation domain
BiFC: Bimolecular Fluorescence Complementation
MT: Microtubule
CDELS: Cell Division Enabled Leaf System
