# Supplementary material for: Functional characterization of D-type cyclins involved in cell division in rice
Source: BMC Plant Biol. 2024 Mar 1;24:157. doi: 10.1186/s12870-024-04828-9 (PMC10905880; doi:10.1186/s12870-024-04828-9)
Supplement: Supplementary file 1 — Supplementary Material 1 [file 12870_2024_4828_MOESM1_ESM.docx]

Supplementary Material

# Supplementary Figures and Tables

## Supplementary Figures


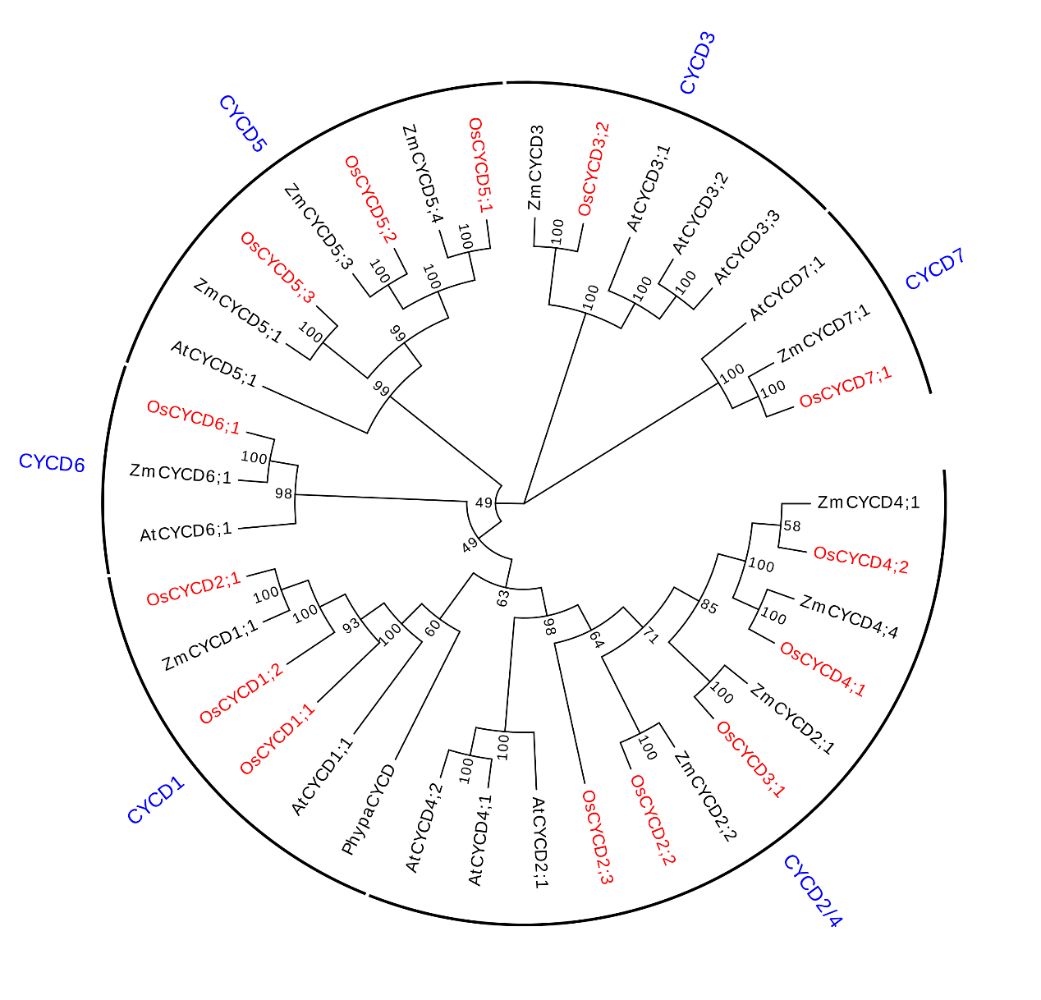


**Fig. S1** Phylogenetic analysis of CYCD gene family in rice, Arabidopsis, and maize. Boostrap values indicate the divergence of each branch.Sequence data can be found at NCBI databases (www.ncbi.nlm.nih.gov): OsCYCD1;1 (Os06g0236600), OsCYCD1;2 (Os08g0421100), OsCYCD2;1 (Os09g0382300), OsCYCD2;2 (Os07g0620800), OsCYCD2;3 (Os03g0392000), OsCYCD3;1 (Os06g0217900), OsCYCD3;2 (Os09g0111100), OsCYCD4;1 (Os09g0466100), OsCYCD4;2(Os08g0479300), OsCYCD5;1 (Os03g0617500), OsCYCD5;2 (Os12g0588800), OsCYCD5;3 (Os03g0203800), OsCYCD6;1 (Os07g0556000), OsCYCD7;1 (Os11g0706801), AtCYCD1;1 (At1g70210), AtCYCD2;1 (At2g22490), AtCYCD3;1 (At4g34160), AtCYCD3;2 (At5g67260), AtCYCD3;3 (At3g50070), AtCYCD4;1 (At5g65420), AtCYCD4;2 (At5g10440), AtCYCD5;1 (At4g37630), AtCYCD6;1 (At4g03270), AtCYCD7;1 (At5g02110), ZmCYCD1;1 (Zm00001d020353), ZmCYCD2;1 (Zm00001d052185), ZmCYCD2;2 (Zm00001d022175), ZmCYCD3 (Zm00001d005293), ZmCYCD4;1 (Zm00001d031837), ZmCYCD4;4 (Zm00001d005928), ZmCYCD5;1 (Zm00001d048143), ZmCYCD5;3 (Zm00001d041345), ZmCYCD5;4 (Zm00001d033330), ZmCYCD6;1 (Zm00001d021745), and ZmCYCD7;1 (Zm00001d048594).


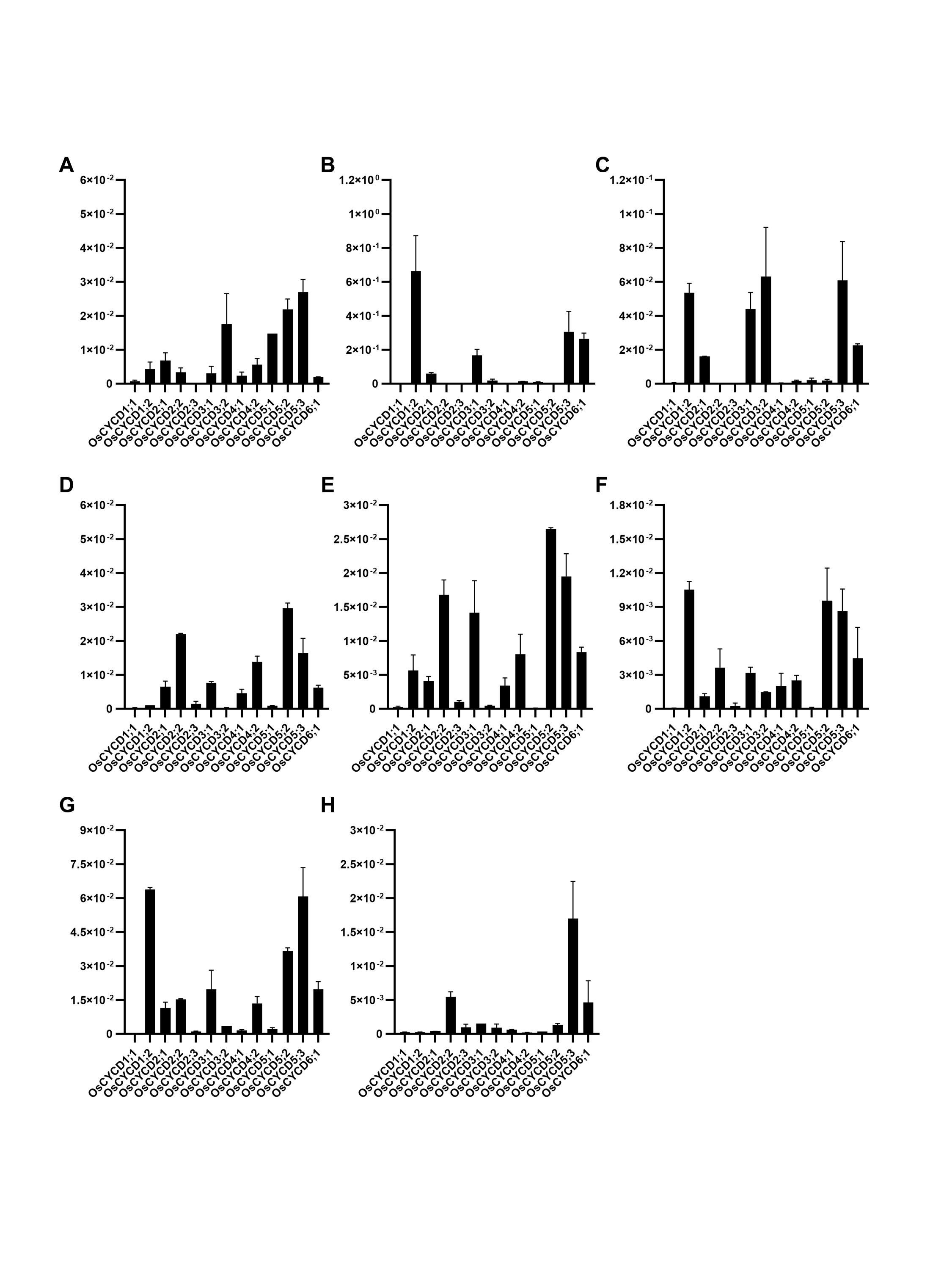


**Fig. S2** The expression level of OsCYCDs in rice tissues including root (A), young leaf (B), mature leaf (C), young inflorescence (< 2 cm, D), ( 2 - 5 cm, E), (> 5 cm, F), spikelet 1 day after fertilization (G), and 5 DAFs seeds (H).


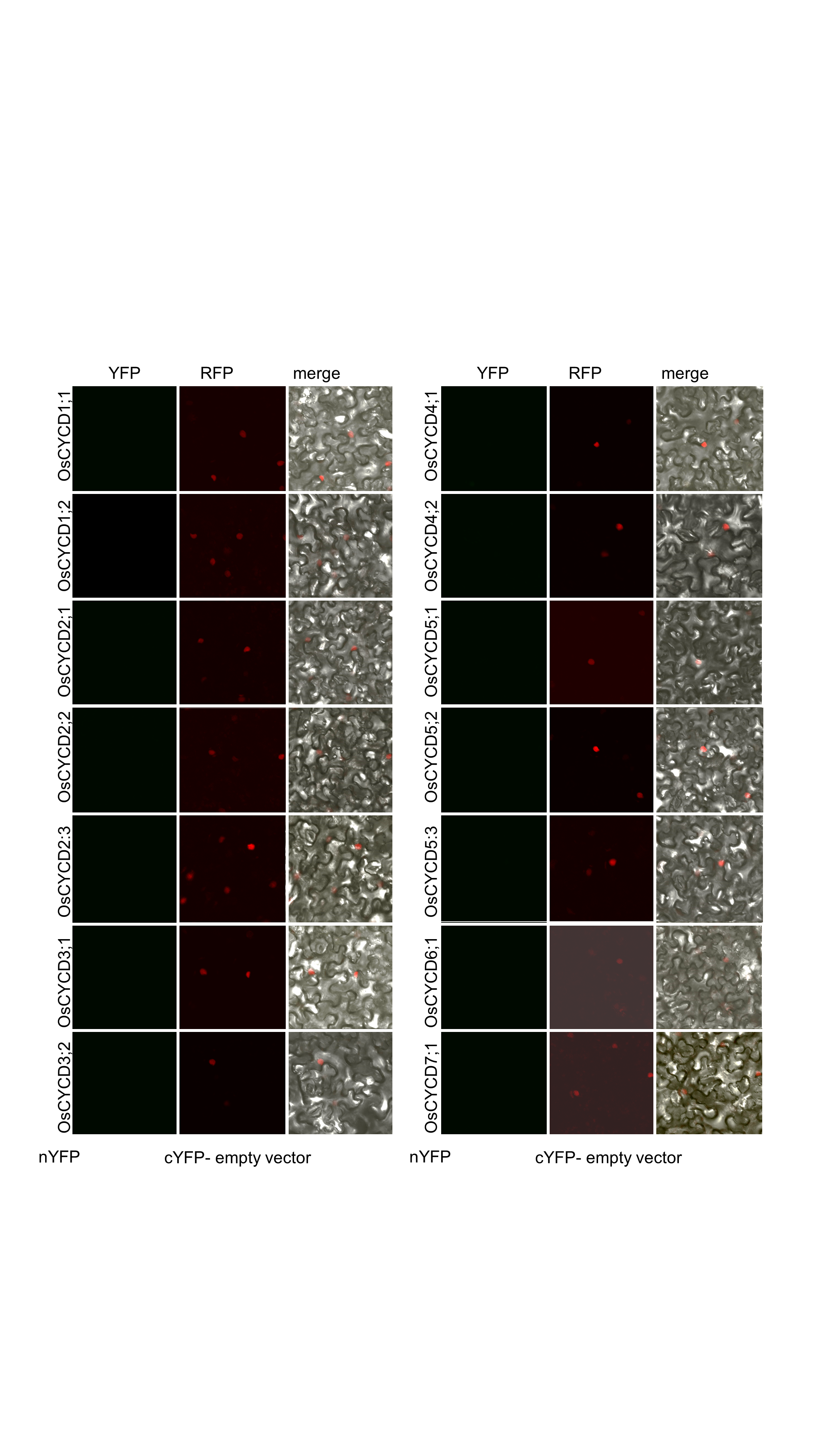


**Fig. S3** The fuorescence signals of *Nicotiana benthamiana* pavement cells co-expressing p35S::OsCYCD-nYFP plasmids and empty p35S::cYFP vectors as negative control for BiFC assay. Histone H1.2-RFP acts as a nuclear marker.


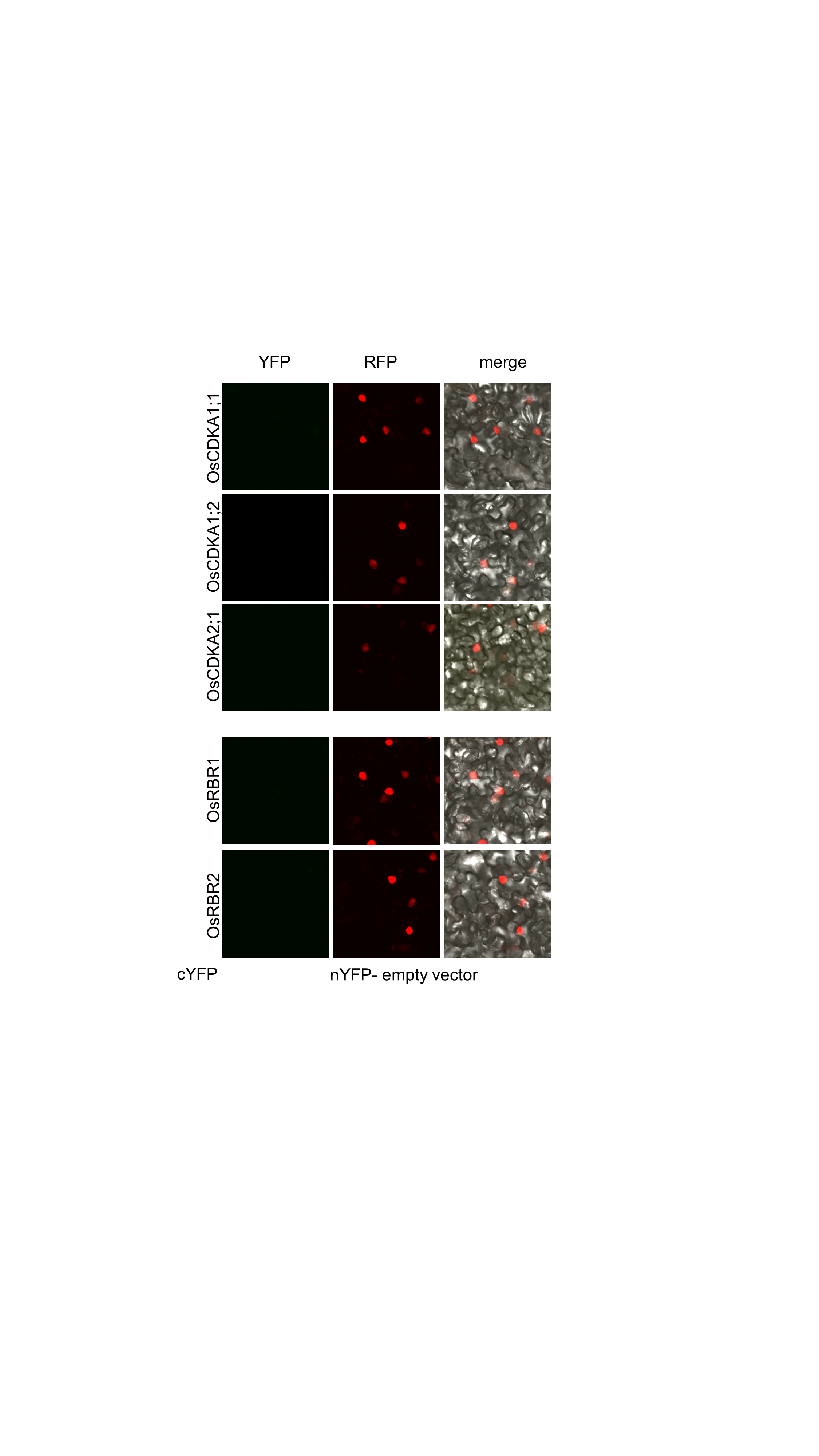


**Fig. S4** The fuorescence signals of *Nicotiana benthamiana* pavement cells co-expressing empty p35S::nYFP vectors and p35S::OsRBR-cYFP or p35S::OsCDKA-cYFP plasmids as negative control for BiFC assay. Histone H1.2-RFP acts as a nuclear marker.


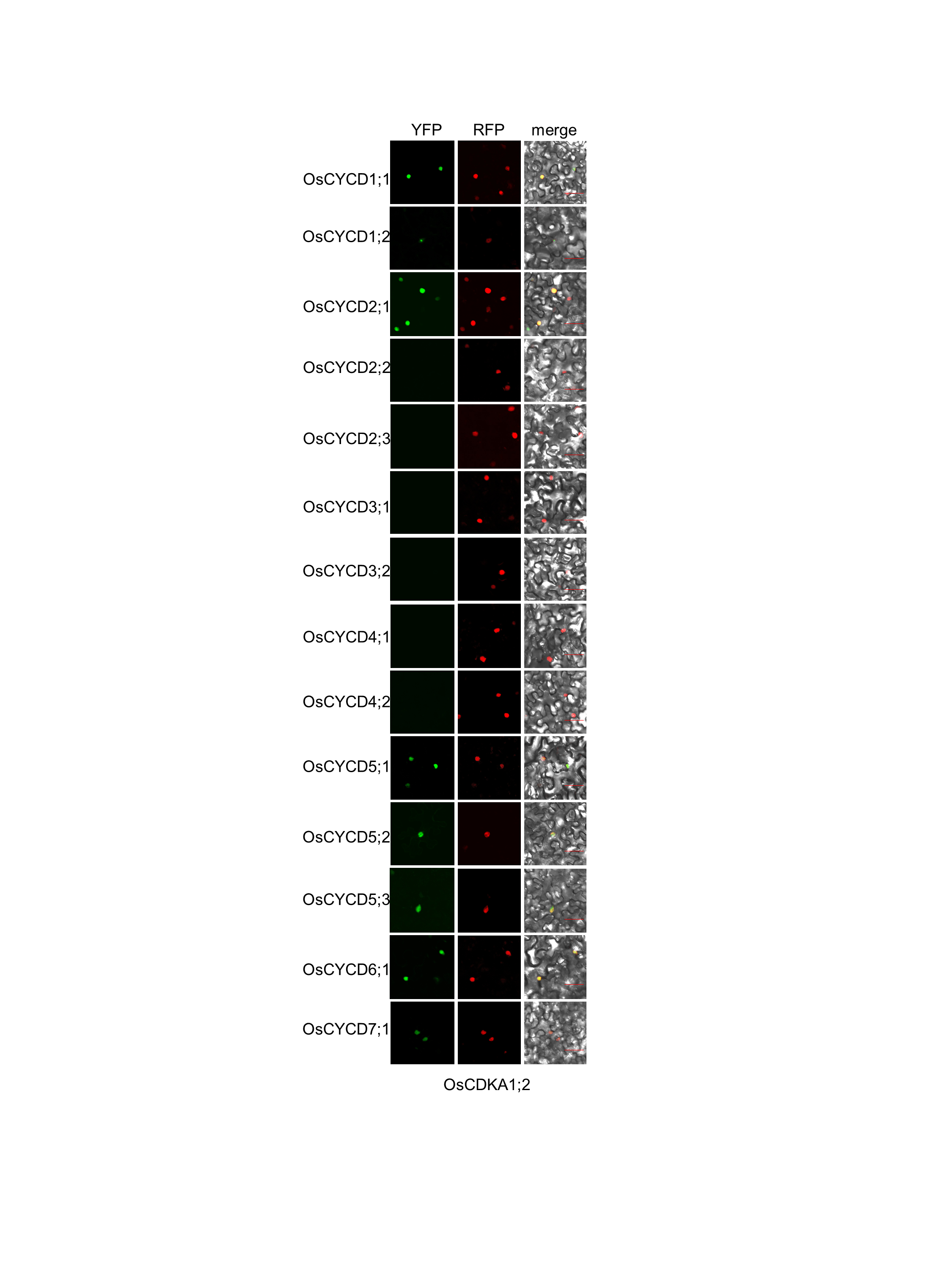


**Fig. S5** BiFC assay of the interaction between OsCYCDs and OsCDKA1;2. Histone H1.2-RFP acts as a nuclear marker. Bar = 50μm.


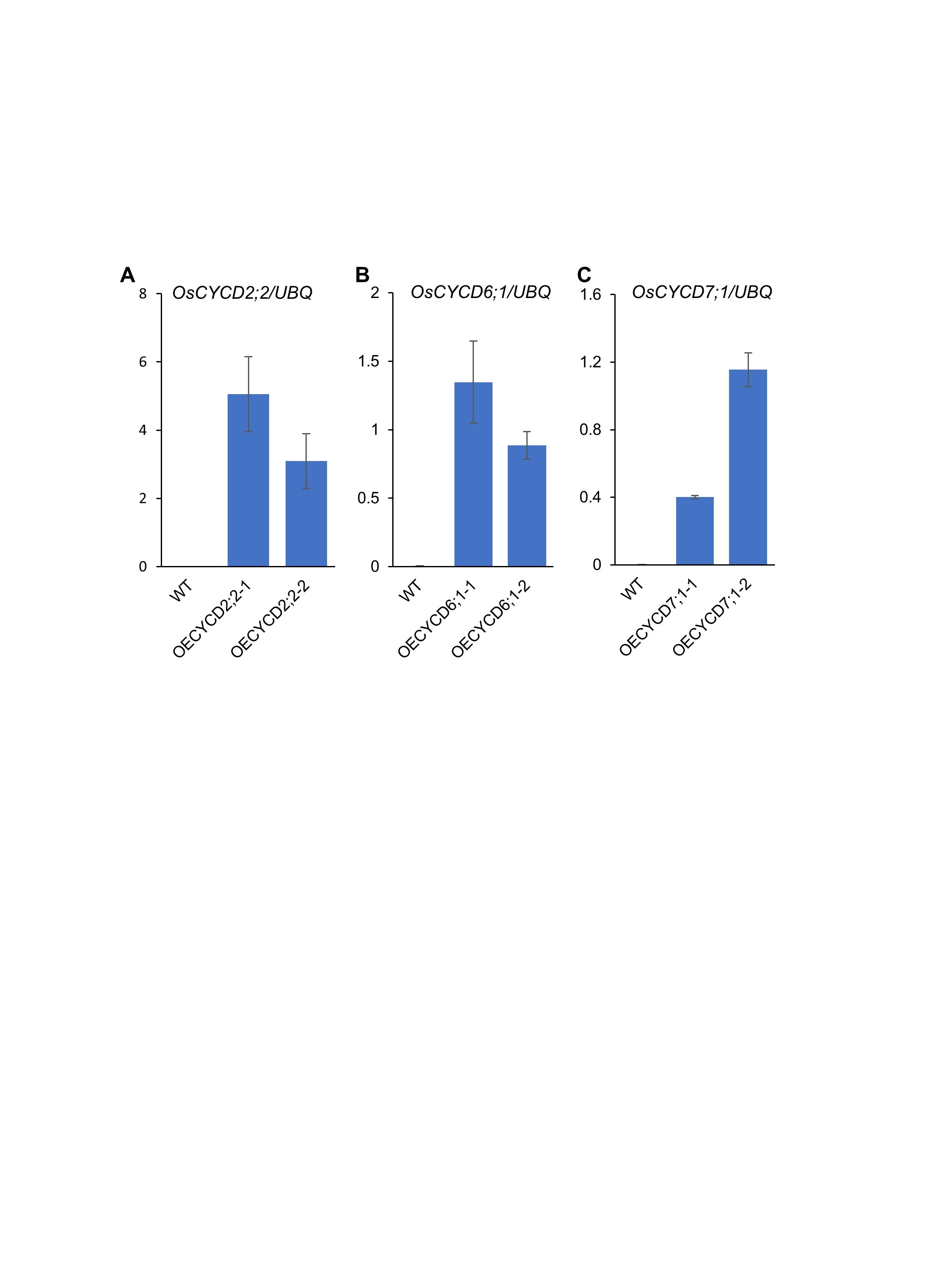


**Fig. S6** The relative expression level of OsCYCDs in corresponding overexpressing plants. **(A)** The transcription level of OsCYCD2;2 in OE-OsCYCD2;2 plants. **(B)** The transcription level of OsCYCD6;1 in OE-OsCYCD6;1 plants. **(C)** The transcription level of OsCYCD7;1 in OE-OsCYCD7;1 plants.

**Table S1** The sequences of primers used for qPCR

| Primers | sequence |
| --- | --- |
| RT-1;1-F | CGCTTGTAAGGTTGGCTCCTCTGGAAG |
| RT-1;1-R | GCTCCTGTGCGACATGGCTGC |
| RT-1;2-F | GATTGGAGGCTTCGATCGATAACACCC |
| RT-1;2-R | ATCTCACTGGAGGCGCATAGCAC |
| RT-2;1-F | GCTGCCAAGATGGAGGAAACCCTAG |
| RT-2;1-R | TCGATGAACGTGAAAGGCGTAACCG |
| RT-2;2-F | GCTGTGCTTGCTGAGAATCAGTTTCTTG |
| RT-2;2-R | CTGTGTGGAACTGAAGAGCTTGCATTG |
| RT-2;3-F | GCACCATGAAAGCAACTGTGTTCC |
| RT-2;3-R | CTCCCCACATTGTCCTTATCTACAGGG |
| RT-3;1-F | ATCGCGGCCAGTGTAGCTCTAGTAG |
| RT-3;1-R | GCCGGCTGATTGGCGCATAATG |
| RT-3;2-F | GCTGATGCCCGATTGGAGGT |
| RT-3;2-R | GCCATCTCATCCTTGGGGGCATC |
| RT-4;1-F | GTGCATAGCTAGAGGAACAGAATGCTT |
| RT-4;1-R | GGCAATGCGACATCCTCTCCTTATTTA |
| RT-4;2-F | CGCATAGCAGCAGGAACTGGATTC |
| RT-4;2-R | CAACACCCTCCCCTTATCTACATGGG |
| RT-5;1-F | TGGATCCTCGAGACGCGAG |
| RT-5;1-R | ATCACCGACCTGTCGATGC |
| RT-5;2-F | CAGCTGAAGCTGGAAGTGTG |
| RT-5;2-R | GCAGGCATGTACATTCTCCTTGT |
| RT-5;3-F | CGCCTCGATCAAAGTCATTAGCTCG |
| RT-5;3-R | CACATGCCCGGTGTCTAGTTGC |
| RT-6;1-F | CTTCCTCGGCTTCTTTCTCTCCGC |
| RT-6;1-R | GAGGGGGAGAACTCCGCCATC |
| RT-7;1-F | TCCTCCGCTCCCTCTTGGATC |
| RT-7;1-R | CACCTCATGGTCCGTCTGGGAGAAA |
| UBQ-F | ACCACTTCGACCGCCACTACT |
| UBQ-R | ACGCCTAAGCCTGCTGGTT |

**Table S2** The sequences of primers used for Y2H vectors construction

| Primers | sequence |
| --- | --- |
| AD-CDKA1;1-F | TGGCCATGGAGGCCAGTGAATTCATGGAGCAGGTGAGCGC |
| AD-CDKA1;1-R | ATCTGCAGCTCGAGCTCGATGGATCCTCATTGTACCATCTCAAGGTCCTTGAAGT |
| AD-CDKA1;2-F | TGGCCATGGAGGCCAGTGAATTCATGGCGCCCCGGC |
| AD-CDKA1;2-R | ATCTGCAGCTCGAGCTCGATGGATCCTCATTGTACCATCTCAAGGTCCTTGAAGT |
| AD-CDKA2;1-F | TGGCCATGGAGGCCAGTGAATTCATGCCACAAGCCCAACCC |
| AD-CDKA2;1-R | ATCTGCAGCTCGAGCTCGATGGATCCCTACGCCACTTCCAGGTCCT |
| AD-RBR1-F | TGGCCATGGAGGCCAGTGAATTCATGGAGGGTGCCGCG |
| AD-RBR1-R | ATCTGCAGCTCGAGCTCGATGGATCCCTAGCAGTCTGGCTGCTCCA |
| AD-RBR2-F | TGGCCATGGAGGCCAGTGAATTCATGGCGTCGCAGCCT |
| AD-RBR2-R | ATCTGCAGCTCGAGCTCGATGGATCCTCAAGAGTCCGTTTTGCTCTTCTTTGAG |
| BD-CYCD1;1-F | GCATATGGCCATGGAGGCCGAATTCATGATGTACAAGCATGTCGGAGAC |
| BD-CYCD1;1-R | CCGCTGCAGGTCGACGGATCCTCAATCTTCTCCAAAATGGCCATCAAGT |
| BD-CYCD1;2-F | GCATATGGCCATGGAGGCCGAATTCATGGGGGCGCCGG |
| BD-CYCD1;2-R | CCGCTGCAGGTCGACGGATCCTCACATCTTCCTCCTCTTTGATGGGG |
| BD-CYCD2;1-F | GCATATGGCCATGGAGGCCGAATTCATGCCGGCCGACGAC |
| BD-CYCD2;1-R | CCGCTGCAGGTCGACGGATCCTCACCCAGCAAAAACATCTGTATTTTTACCA |
| BD-CYCD2;2-F | GCATATGGCCATGGAGGCCGAATTCATGGGTGTTCTTTGCTTCGGC |
| BD-CYCD2;2-R | CCGCTGCAGGTCGACGGATCCTCAGATTGGTGTTGTGTTTAATCTCCTCCT |
| BD-CYCD2;3-F | GCATATGGCCATGGAGGCCGAATTCATGGGGTTCCTCTGCCG |
| BD-CYCD2;3-R | CCGCTGCAGGTCGACGGATCCCTACTCGCCGCCGGC |
| BD-CYCD3;1-F | GCATATGGCCATGGAGGCCGAATTCATGGCACCGAGCTTTGATTTTG |
| BD-CYCD3;1-R | CCGCTGCAGGTCGACGGATCCTCATCTGCAAATTCTCCTCCTTTTGCT |
| BD-CYCD3;2-F | GCATATGGCCATGGAGGCCGAATTCATGGCTTTCGCCACGC |
| BD-CYCD3;2-R | CCGCTGCAGGTCGACGGATCCTCATGCGGCGTACGGCG |
| BD-CYCD4;1-F | GCATATGGCCATGGAGGCCGAATTCATGGCAGCTCTGACGAGC |
| BD-CYCD4;1-R | CCGCTGCAGGTCGACGGATCCTCATCTGCTAATCTTCCTCCTCTTGCT |
| BD-CYCD4;2-F | CCGCTGCAGGTCGACGGATCCTCACCTGCTAAGCTTCCTCCTCT |
| BD-CYCD4;2-R | GCATATGGCCATGGAGGCCGAATTCATGGCTCCGAGCTCGT |
| BD-CYCD5;1-F | GCATATGGCCATGGAGGCCGAATTCATGGAGGCAGAGGACGAGTAC |
| BD-CYCD5;1-R | CCGCTGCAGGTCGACGGATCCTCATCGGAGGATGCCTCCCG |
| BD-CYCD5;2-F | GCATATGGCCATGGAGGCCGAATTCATGTCGATGGAGGAGGCGG |
| BD-CYCD5;2-R | CCGCTGCAGGTCGACGGATCCTCAACGGATTCGCGGCG |
| BD-CYCD5;3-F | GCATATGGCCATGGAGGCCGAATTCATGGGGGACGCCTCG |
| BD-CYCD5;3-R | CCGCTGCAGGTCGACGGATCCCTACTGGCGCTGAGGCG |
| BD-CYCD6;1-F | GCATATGGCCATGGAGGCCGAATTCATGGACATGGCGACGGG |
| BD-CYCD6;1-R | CCGCTGCAGGTCGACGGATCCTCACTCATCAGGGCCGCC |
| BD-CYCD7;1-F | GCATATGGCCATGGAGGCCGAATTCATGGATGATGACGACGATACTAGTTTCAA |
| BD-CYCD7;1-R | CCGCTGCAGGTCGACGGATCCTCAAATTGATCTGGCTTTTAATCTGCCG |

**Table S3** The sequences of primers used for BiFC vectors construction

| Primers | sequence |
| --- | --- |
| YC-CDKA1;1-F | CGACTCTAGGAGCTCGGTACCCGGGATGGAGCAGGTGAGCGC |
| YC-CDKA1;1-R | ATCTGGAACATCGTATGGGTACATACTAGTTTGTACCATCTCAAGGTCCTTGAAG |
| YC-CDKA1;2-F | CGACTCTAGGAGCTCGGTACCCGGGATGGCGCCCCGGC |
| YC-CDKA1;2-R | ATCTGGAACATCGTATGGGTACATACTAGTTTGTACCATCTCAAGGTCCTTGAAG |
| YC-CDKA2;1-F | CGACTCTAGGAGCTCGGTACCCGGGATGCCACAAGCCCAACCC |
| YC-CDKA2;1-R | ATCTGGAACATCGTATGGGTACATACTAGTCGCCACTTCCAGGTCCTTGA |
| YC-RBR1-F | CGACTCTAGGAGCTCGGTACCCGGGATGGAGGGTGCCGCG |
| YC-RBR1-R | ATCTGGAACATCGTATGGGTACATACTAGTGCAGTCTGGCTGCTCCAAT |
| YC-RBR2-F | CGACTCTAGGAGCTCGGTACCCGGGATGGCGTCGCAGCCT |
| YC-RBR2-R | ATCTGGAACATCGTATGGGTACATACTAGTAGAGTCCGTTTTGCTCTTCTTTGAG |
| YN-CYCD1;1-F | GTTGATTTCTGAGGAGGATCTTCCCGGGATGTACAAGCATGTCGGAGACG |
| YN-CYCD1;1-R | CAGGGCATGCCTGCAGGTCGACTCAATCTTCTCCAAAATGGCCATCAAGT |
| YN-CYCD1;2-F | GTTGATTTCTGAGGAGGATCTTCCCGGGGGGGCGCCGGCG |
| YN-CYCD1;2-R | CAGGGCATGCCTGCAGGTCGACTCACATCTTCCTCCTCTTTGATGGGG |
| YN-CYCD2;1-F | GTTGATTTCTGAGGAGGATCTTCCCGGGCCGGCCGACGAC |
| YN-CYCD2;1-R | CAGGGCATGCCTGCAGGTCGACTCACCCAGCAAAAACATCTGTATTTTTACCA |
| YN-CYCD2;2-F | GTTGATTTCTGAGGAGGATCTTCCCGGGGGTGTTCTTTGCTTCGGC |
| YN-CYCD2;2-R | CAGGGCATGCCTGCAGGTCGACTCAGATTGGTGTTGTGTTTAATCTCCTCCT |
| YN-CYCD2;3-F | GTTGATTTCTGAGGAGGATCTTCCCGGGGGGTTCCTCTGCCGC |
| YN-CYCD2;3-R | CAGGGCATGCCTGCAGGTCGACCTACTCGCCGCCGGC |
| YN-CYCD3;1-F | GTTGATTTCTGAGGAGGATCTTCCCGGGGCACCGAGCTTTGATTTTGC |
| YN-CYCD3;1-R | CAGGGCATGCCTGCAGGTCGACTCATCTGCAAATTCTCCTCCTTTTGCT |
| YN-CYCD3;2-F | GTTGATTTCTGAGGAGGATCTTCCCGGGGCTTTCGCCACGCTC |
| YN-CYCD3;2-R | CAGGGCATGCCTGCAGGTCGACTCATGCGGCGTACGGCG |
| YN-CYCD4;1-F | GTTGATTTCTGAGGAGGATCTTCCCGGGGCAGCTCTGACGAGCT |
| YN-CYCD4;1-R | CAGGGCATGCCTGCAGGTCGACTCATCTGCTAATCTTCCTCCTCTTGCT |
| YN-CYCD4;2-F | GTTGATTTCTGAGGAGGATCTTCCCGGGGCTCCGAGCTCGTCG |
| YN-CYCD4;2-R | CAGGGCATGCCTGCAGGTCGACTCACCTGCTAAGCTTCCTCCTCT |
| YN-CYCD5;1-F | GTTGATTTCTGAGGAGGATCTTCCCGGGGAGGCAGAGGACGAGTACT |
| YN-CYCD5;1-R | CAGGGCATGCCTGCAGGTCGACTCATCGGAGGATGCCTCCCG |
| YN-CYCD5;2-F | GTTGATTTCTGAGGAGGATCTTCCCGGGTCGATGGAGGAGGCGG |
| YN-CYCD5;2-R | CAGGGCATGCCTGCAGGTCGACTCAACGGATTCGCGGCG |
| YN-CYCD5;3-F | GTTGATTTCTGAGGAGGATCTTCCCGGGGGGGACGCCTCGG |
| YN-CYCD5;3-R | CAGGGCATGCCTGCAGGTCGACCTACTGGCGCTGAGGCG |
| YN-CYCD6;1-F | GTTGATTTCTGAGGAGGATCTTCCCGGGGACATGGCGACGGGG |
| YN-CYCD6;1-R | CAGGGCATGCCTGCAGGTCGACTCACTCATCAGGGCCGCC |
| YN-CYCD7;1-F | GTTGATTTCTGAGGAGGATCTTCCCGGGGATGATGACGACGATACTAGTTTC |
| YN-CYCD7;1-R | CAGGGCATGCCTGCAGGTCGACTCAAATTGATCTGGCTTTTAATCTGCCG |

**Table S4** The sequences of primers used for overexpression vectors construction

| Primers | sequence |
| --- | --- |
| OV-1;1-F | TCGCGAGCTCGGTACCATGATGTACAAGCATGTCGGAGACG |
| OV-1;1-R | GCAGGTCGACTCTAGAGGATCCTCAATCTTCTCCAAAATGGCCATCAAGT |
| OV-1;2-F | TCGCGAGCTCGGTACCATGGGGGCGCCGGCGACCGC |
| OV-1;2-R | GCAGGTCGACTCTAGAGGATCCTCACATCTTCCTCCTCTTTGATGGGGG |
| OV-2;1-F | TCGCGAGCTCGGTACCATGCCGGCCGACGACGACGA |
| OV-2;1-R | GCAGGTCGACTCTAGAGGATCCTCACCCAGCAAAAACATCTGTATTTTTAC |
| OV-2;2-F | TCGCGAGCTCGGTACCATGGGTGTTCTTTGCTTCGG |
| OV-2;2-R | GCAGGTCGACTCTAGAGGATCCTCAGATTGGTGTTGTGTTTAATCTCC |
| OV-2;3-F | TCGCGAGCTCGGTACCATGGGGTTCCTCTGCCGCC |
| OV-2;3-R | GCAGGTCGACTCTAGAGGATCCCTACTCGCCGCCGGCCTGCTCC |
| OV-3;1-F | TCGCGAGCTCGGTACCATGGCACCGAGCTTTGATTTTG |
| OV-3;1-R | GCAGGTCGACTCTAGAGGATCCTCATCTGCAAATTCTCCTCC |
| OV-3;2-F | TCGCGAGCTCGGTACCATGGCTTTCGCCACGCTCTTTG |
| OV-3;2-R | GCAGGTCGACTCTAGAGGATCCTCATGCGGCGTACGGCGGCC |
| OV-4;1-F | TCGCGAGCTCGGTACCATGGCAGCTCTGACGAGCTACG |
| OV-4;1-R | TGCAGGTCGACTCTAGAGGATCCTCATCTGCTAATCTTCCTCC |
| OV-4;2-F | TCGCGAGCTCGGTACCATGGCTCCGAGCTCGTCGTCC |
| OV-4;2-R | GCAGGTCGACTCTAGAGGATCCTCACCTGCTAAGCTTCCTCC |
| OV-5;1-F | TCGCGAGCTCGGTACCATGGAGGCAGAGGACGAGTACTC |
| OV-5;1-R | GCAGGTCGACTCTAGAGGATCCTCATCGGAGGATGCCTCCCGG |
| OV-5;2-F | TCGCGAGCTCGGTACCATGTCGATGGAGGAGGCGGAGGAG |
| OV-5;2-R | GCAGGTCGACTCTAGAGGATCCTCAACGGATTCGCGGCGGCTC |
| OV-5;3-F | TCGCGAGCTCGGTACCATGGGGGACGCCTCGGCATCC |
| OV-5;3-R | GCAGGTCGACTCTAGAGGATCCCTACTGGCGCTGAGGCGAGTGCAACC |
| OV-6;1-F | TCGCGAGCTCGGTACCATGGACATGGCGACGGGGGCG |
| OV-6;1-R | GCAGGTCGACTCTAGAGGATCCTCACTCATCAGGGCCGCCTAC |
| OV-7;1-F | TCGCGAGCTCGGTACCATGGATGATGACGACGATAC |
| OV-7;1-R | GCAGGTCGACTCTAGAGGATCCTCAAATTGATCTGGCTTTTAATC |

**Table S5** The basic characteristics of CYCD proteins in rice

| Gene | Locus | CDS length  (bp) | amino acids | isoelectric point | molecular weight（KD） |
| --- | --- | --- | --- | --- | --- |
| *OsCYCD1;1* | LOC_Os06g12980 | 1092 | 363 | 5.01 | 39.25 |
| *OsCYCD1;2* | LOC_Os08g32540 | 1065 | 354 | 4.68 | 38.57 |
| *OsCYCD2;1* | LOC_Os09g21450 | 690 | 229 | 4.58 | 25.40 |
| *OsCYCD2;2* | LOC_Os07g42860 | 1071 | 356 | 5.04 | 39.18 |
| *OsCYCD2;3* | LOC_Os03g27420 | 1218 | 405 | 4.65 | 44.66 |
| *OsCYCD3;1* | LOC_Os06g11410 | 1029 | 342 | 4.72 | 38.28 |
| *OsCYCD3;2* | LOC_Os09g02360 | 1095 | 364 | 4.89 | 38.34 |
| *OsCYCD4;1* | LOC_Os09g29100 | 1071 | 356 | 4.82 | 38.93 |
| *OsCYCD4;2* | LOC_Os08g37390 | 1152 | 383 | 4.94 | 41.66 |
| *OsCYCD5;1* | LOC_Os03g42070 | 1104 | 367 | 4.75 | 38.72 |
| *OsCYCD5;2* | LOC_Os12g39830 | 1098 | 365 | 4.67 | 39.27 |
| *OsCYCD5;3* | LOC_Os03g10650 | 1038 | 345 | 4.99 | 38.06 |
| *OsCYCD6;1* | LOC_Os07g37010 | 963 | 320 | 5.48 | 34.17 |
| *OsCYCD7;1* | LOC_Os11g47950 | 963 | 320 | 5.78 | 35.98 |
